# Novel wave intensity analysis of arterial pulse wave propagation accounting for peripheral reflections

**DOI:** 10.1002/cnm.2602

**Published:** 2013-10-16

**Authors:** Jordi Alastruey, Anthony A E Hunt, Peter D Weinberg

**Affiliations:** 1 Department of Biomedical Engineering, Division of Imaging Sciences and Biomedical Engineering, King's College London, King's Health Partners, St. Thomas' HospitalLondon, SE1 7EH, U.K.; 2 Department of Bioengineering, Imperial CollegeLondon, SW7 2AZ, U.K.

**Keywords:** haemodynamics, pulse wave propagation, wave intensity analysis, one-dimensional modelling, Windkessel effect, *PU*–loop method, systemic circulation

## Abstract

We present a novel analysis of arterial pulse wave propagation that combines traditional wave intensity analysis with identification of Windkessel pressures to account for the effect on the pressure waveform of peripheral wave reflections. Using haemodynamic data measured *in vivo* in the rabbit or generated numerically in models of human compliant vessels, we show that traditional wave intensity analysis identifies the timing, direction and magnitude of the predominant waves that shape aortic pressure and flow waveforms in systole, but fails to identify the effect of peripheral reflections. These reflections persist for several cardiac cycles and make up most of the pressure waveform, especially in diastole and early systole. Ignoring peripheral reflections leads to an erroneous indication of a reflection-free period in early systole and additional error in the estimates of (i) pulse wave velocity at the ascending aorta given by the *PU*–loop method (9.5% error) and (ii) transit time to a dominant reflection site calculated from the wave intensity profile (27% error). These errors decreased to 1.3% and 10%, respectively, when accounting for peripheral reflections. Using our new analysis, we investigate the effect of vessel compliance and peripheral resistance on wave intensity, peripheral reflections and reflections originating in previous cardiac cycles.

## 1 INTRODUCTION

Blood pressure and flow waveforms in systemic arteries carry valuable information for the diagnosis and treatment of cardiovascular disease and play a significant role in clinical conditions such as hypertension. The waveforms result from a complex ventricular-vascular interaction involving cardiac contraction, impedance of large and medium-sized distensible arteries and resistance of smaller arteries and arterioles. Blood behaves as an incompressible fluid in arteries, which distend to accommodate the sudden increase in blood volume delivered by cardiac contraction. When elastic energy stored in the distended arterial walls is released, arteries contract. The regular expansion and contraction of arteries (the *pulse*) that follows cardiac contraction propagates in the form of *pulse waves*. These produce continuous changes in blood pressure and flow that can be studied as pressure and flow *wavefronts* (infinitesimal changes in pressure and flow)‡ running forwards and backwards (away from and towards the heart, respectively), with backward wavefronts originating from reflected forward wavefronts at sites of vascular impedance mismatch.

**Figure 1:**
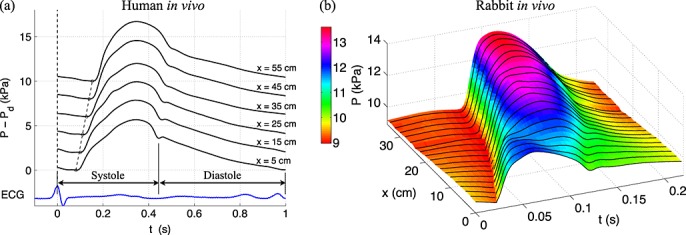
Blood pressure (*P*) waveform measured *in vivo* along the human (left, modified from 1) and rabbit (right) aortas. (a) Measurements were made every 10 cm down the aorta starting approximately 5 cm from the aortic valve. Each waveform is an ensemble average of continuous pressure measurements over 1 min using the peak of the R-wave of the electrocardiogram as the reference time. The circles indicate the time of the diastolic pressure (*P*_d_) after which pressure increases due to left ventricular ejection of blood. The slope of the dotted lines connecting the circles indicates the pulse wave speed (6.9 m s ^ − 1^) with which the pressure wavefront at the start of cardiac contraction propagates down the aorta. Systole is the phase of the cardiac cycle when the heart muscle contracts, and diastole is the phase when the heart muscle relaxes. (b) Measurements were made every 1 cm starting approximately 1 cm from the aortic valve, as described in Section 2.1. Each waveform is an ensemble average of ten continuous cardiac cycles using the foot of the flow waveform in the aortic root as the reference time.

Figure 1 shows typical blood pressure waveforms measured *in vivo* along human (left) and rabbit (right) aortas, from the root to the aorto-iliac bifurcation, under normal conditions. The slope of the line joining the feet of these waveforms shows clearly that the pressure wavefront originated at the start of cardiac contraction propagates away from the heart; the measured space-averaged speed is 6.9 m s ^ − 1^ in the human and 6.1 m s ^ − 1^ in the rabbit. Thus, during a typical cardiac cycle, which takes about 1 s in the human and 0.25 s in the rabbit, a pulse wave has sufficient time to travel from the heart to the arterial vasculature and back multiple times.

Several studies have used wave intensity analysis (WIA) to investigate the role of wave reflections in shaping *in vivo* pressure and flow waveforms in systemic arteries 2–11, including the coronary circulation 12–14. Given simultaneous measurements of blood pressure and flow velocity with time at an arbitrary location in the arterial network, we can calculate the local pulse wave velocity (PWV) and apply WIA to quantify the timing, direction and magnitude of the predominant waves that shape the pressure and velocity waveforms 15,16. Accurate estimation of PWV is not only important for WIA but is also clinically relevant, because PWV is an important predictor of cardiovascular events 17.

Numerical modelling has been used to assess the following: (i) the ability of WIA to quantify reflection coefficients 18; (ii) haemodynamic information provided by WIA in a model of aortic coarctation 19 and the fetal circulation 20; (iii) a modified WIA based on the reservoir-wave separation 21; and (iv) the performance of several methods for PWV calculation 22–25. Numerically generated pressure and flow waveforms are free of measurement errors, and the theoretical values of haemodynamic properties that affect waveforms (e.g. PWV, location of reflection sites, and magnitude of reflected waves produced) are available for comparison with corresponding estimates given by WIA and methods of calculating PWV.

In the present work, we used pressure and flow velocity waveforms measured *in vivo* in the rabbit or generated numerically in several models of human compliant vessels to (i) show the inability of traditional WIA to identify the important role of peripheral reflections in shaping the pressure waveform; (ii) test the accuracy of the modified *PU*–loop method of calculating PWV proposed by Mynard *et al.* 24, which accounts for peripheral reflections originating in previous cardiac cycles; and (iii) propose a new analysis of arterial pulse wave propagation to study the predominant waves that shape pressure and flow waveforms during systole and the contribution to the pressure waveform, over the whole cardiac cycle, of wave reflections originating in previous cardiac cycles, vessel compliances, peripheral resistances, outflow pressures and the flow at the root. We used our new analysis to study the effects of vessel stiffness and peripheral resistance on numerically-generated aortic pressure and flow waveforms.

We generated all numerical data using the nonlinear one-dimensional (1-D) formulation of blood flow in compliant vessels, because WIA is derived from this formulation and, hence, 1-D model pressure and velocity waveforms provide an ideal mathematical framework for our study. Several comparisons against *in vivo* 26–29, *in vitro* 30–34 and 3-D numerical 35 data have shown the ability of the 1-D formulation to capture the main features of pressure and flow waveforms in large human arteries. The nomenclature and abbreviations used in this paper are listed in the supplementary material.

## 2 METHODS

We first describe the *in vivo* (Section 2.1) and numerical (Section 2.2) pressure and flow waveforms used in our work. Next, we summarise the mathematical formulation of traditional WIA (Section 2.3) and show how we quantified the magnitude and timing of wave reflections (Section 2.4). We then introduce the Windkessel model to study arterial haemodynamics during diastole and account for peripheral reflections in WIA and the *PU*–loop method (Section 2.5).

### 2.1 Rabbit in vivo pressure and flow waveforms

Ten New Zealand white male rabbits (Harlan UK) with the properties shown in Table I were maintained on a standard laboratory diet and housed at 18°C on a 12-h light cycle. The rabbits were pre-medicated with Hypnorm (0.1 ml kg ^ − 1^) intramuscularly and anaesthetised with sodium pentobarbitone (35 mg kg ^ − 1^), administered intravenously via the marginal vein of the right ear. They were then placed in the supine position and artificially ventilated with a Harvard Small Animal Ventilator (with a respiratory rate of 50 breaths min ^ − 1^; inflation pressure set for trough 25 cmH _2_O and peak 50 cmH _2_O). Body temperature was maintained near 39 °C (monitored using a rectal thermistor probe) by placing the rabbits on a heating blanket with a temperature controller (CWE Inc. TC1000). The animal procedures complied with the Animals (Scientific Procedures) Act (1986) and were approved by the Imperial College London local ethical review process.

**Table I:** Properties of the 10 mature rabbits as follows: age, weight (W), crown-to-rump length (C-R), heart rate (HR), cardiac output (CO), mean pressure (*P*_m_), pulse pressure (PP), outflow pressure (*P*_out_), time constant of the decline in pressure during diastole (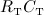) and pulse wave velocity at the aortic root calculated using the foot-to-foot method (*c*_ff_), the traditional *PU*–loop (*c*_PU_) and the modified 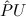
–loop (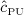), with *ρ* = 1,015 kg m ^ − 3^ in both loop methods.

Rabbit	Age	W	C-R	HR	CO	*P*_m_	PP	*P*_out_	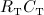	*c*_ff_	*c*_PU_	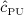
No.	(days)	(kg)	(cm)	(beat/s)	(ml/s)	(kPa)	(kPa)	(kPa)	(s)	(m/s)	(m/s)	(m/s)
1	100	3.22	43	3.7	3.1	8.1	2.9	3.0	0.32	4.3	3.9	4.3
2	114	3.20	41	2.9	2.3	5.2	2.8	2.2	0.37	4.5	4.2	4.4
3	93	2.55	40	4.0	2.4	6.9	2.8	2.3	0.28	4.7	4.2	4.6
4	72	2.29	40	4.5	2.6	6.2	2.2	2.2	0.28	5.1	4.6	5.1
5	92	2.67	40	4.7	4.2	7.7	3.4	2.8	0.18	4.5	4.0	4.5
6	136	2.54	42	3.1	4.1	6.0	2.4	1.3	0.28	3.5	3.0	3.4
7	143	3.00	41	3.4	4.9	8.6	3.1	2.3	0.30	4.5	4.2	4.6
8	114	3.26	43	3.6	4.0	10.5	2.8	4.5	0.32	4.4	4.0	4.4
9	159	3.48	45	4.7	4.4	8.1	2.6	1.7	0.34	4.3	3.9	4.3
10	133	3.13	41	5.2	5.4	8.5	3.0	3.1	0.26	3.9	3.5	3.8
Mean	116	2.93	41.6	4.0	3.7	7.6	2.8	2.5	0.29	4.37	3.96	4.34
SEM	9	0.12	0.5	0.2	0.3	0.5	0.1	0.3	0.02	0.13	0.14	0.14

We calculated *P*_out_ and 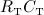 by fitting an exponential function of the form given by Equation (19) to the decline in pressure during diastole, with *T*_0_ the time at the beginning of the exponential fit. The last two rows are mean and standard error of the mean (SEM) for all ten rabbits.

**Figure 2:**
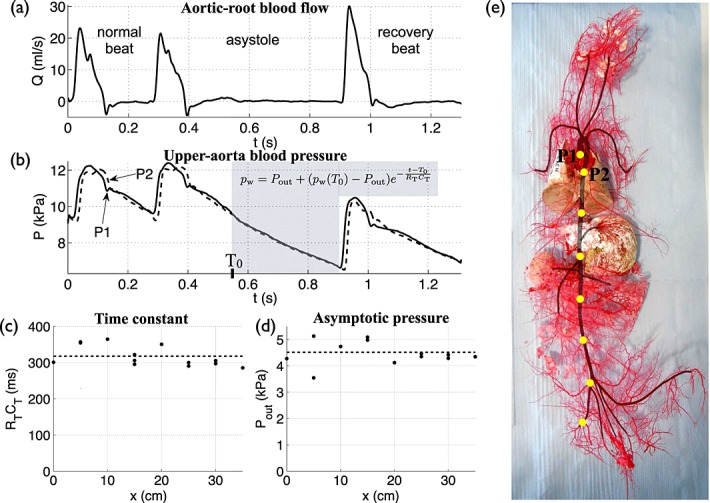
*In vivo* (a) flow rate at the aortic root and (b) pressure at the aortic root (P1) and 5 cm distally in the aorta (P2) with time, measured during an asystole in Rabbit 8. (c) Time constant (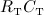) and (d) asymptotic pressure (*P*_out_) calculated along the aorta and iliac artery at 5 cm increments (indicated by yellow dots in (e)). 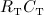 and *P*_out_ were derived from an exponential function (Equation (19)) fitted to the decline in pressure during an asystole (shaded area in (b)). Dashed lines indicate the corresponding average values. (e) Cast of the systemic vasculature of Rabbit 8. The aorta and its main branches are shaded.

For each rabbit, a midline thoracotomy was made and the rib cage was retracted to expose the ascending aorta and place a perivascular blood flow probe (6 mm diameter, type MA6PSB, Transonic Systems Inc.) around it, near the aortic valve. The thoracotomy was closed using clamps. The right femoral artery was exposed at the level between the knee and groin for insertion of pressure measurement wires. Two pressure waveforms were simultaneously measured with the aortic flow waveform, from the aortic root to the iliac artery in 1 cm increments (Figure 1(b)) using a dual sensor Millar Mikro-tip catheter transducer (model SPC-721, size 2.5F, sensors 5 cm apart). The aortic pressure waveforms at the root and 5 cm distally (Figure 2(b)) allowed us to calculate the PWV using the ‘foot-to-foot’ method (*c*_ff_) 25, which we used as the gold standard PWV at the aortic root (Table I). Brief *asystole* (cessation of heart contraction) were induced by gently tapping the left ventricle (LV) (Figure 2(a,b)). All data were acquired at a sampling rate of 1 kHz using the NOTOCORD-hem acquisition software (NOTOCORD Systems, France).

Prior to euthanasia, heparin (2,000 units) was administered intravenously for anticoagulation of the blood in preparation for production of a resin cast of the arterial system (Figure 2(e)). The thorax was reopened and the LV was cannulated with a polythene tube (filled with saline solution: 9 g/L NaCl) via an apical stab wound. The cannula was flushed through with approximately 5 ml saline. Casting resin (Batson's no. 17, Plastic Replica and Corrosion kits, Polyscience, USA, prepared according to the manufacturer's instructions) was infused into the arterial system via the cannula, at a pressure equivalent to the mean arterial pressure that was recorded *in vivo* in the thoracic aorta. Infusion was continued until the casting resin ceased to flow because of setting of the resin; the final perfusion volume was approximately 100 ml of resin. The cast was allowed to cure for at least 12 h; then the carcass was completely submerged in an aqueous solution of potassium hydroxide (25% w/v). The alkaline corrosion process was allowed to continue for 14 days. Then the corrosion solution was removed to reveal the arterial cast, which was submerged in a strong warm solution of detergent (Decon 90, Decon Laboratories Ltd., East Sussex, UK) and left for 24 h. The cast was rinsed gently and thoroughly in water and allowed to dry.

We used the cast to locate accurately the sites of pressure measurements and determine the luminal cross-sectional area of the aortic root, which is required to convert measured flow rate to flow velocity; the latter, combined with simultaneous pressure at the root, allowed WIA.

### 2.2 Human numerical pressure and flow waveforms

**Figure 3:**
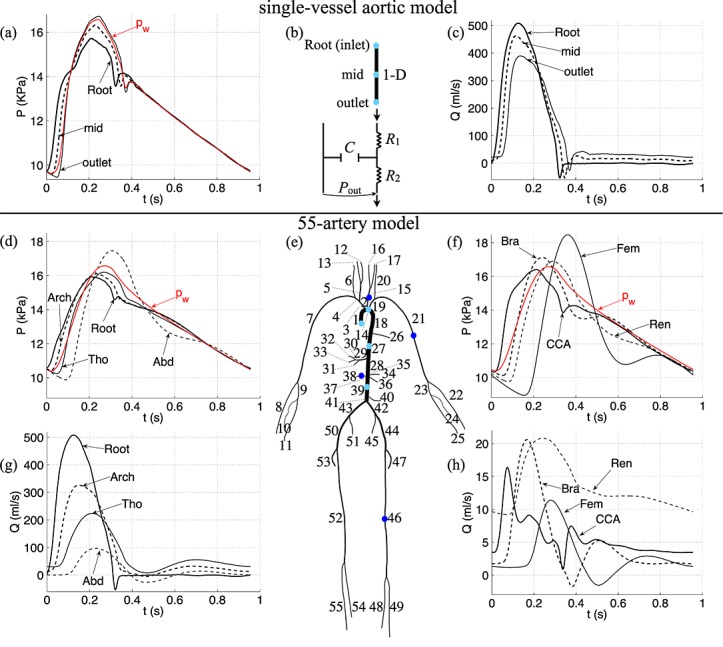
Pressure and flow rate with time, at (a,c) the inlet, midpoint and outlet of a single-vessel model of the human thoracic aorta (b) 35 and (d-h) the aortic root (Root, Segment 1), midpoint of the aortic arch B (Arch, Segment 14), thoracic aorta B (Tho, Segment 27) and abdominal aorta D (Abd, Segment 39), left common carotid (CCA, Segment 15), left brachial (Bra, Segment 21), right renal (Ren, Segment 38) and left femoral (Fem, Segment 46) arteries of a model of the 55 larger systemic arteries in the human (e) 1. They were calculated using the nonlinear purely elastic (a,c) and visco-elastic (d,f,g,h) 1-D equations (1) and (2). At the root of both models, we prescribed a flow rate measured *in vivo* (labelled ‘Root’ in (c,g)). At the outlet of each terminal branch, we coupled a three-element Windkessel model of the perfusion of the microcirculation (b), with *R*_1_ = *Z*_0_ to minimise wave reflections 42. The properties of the aortic model are shown in Table II. The names and properties of the segments in the 55-artery model are shown in Table III. The uniform Windkessel pressure, *p*_w_, given by Equation (17) is shown in red in (a,d,f).

To generate the numerical pressure and flow data for this study, we solved the nonlinear 1-D equations of blood flow in compliant vessels in a single-vessel model of the human thoracic aorta 35 (Figure 3(a,b,c), Table II) and also in a model of the 55 larger systemic arteries in the human 1 (Figure 3(d,e,f,g,h), Table III), using a DG scheme with a spectral/*hp* spatial discretisation 32. These equations can be derived by applying conservation of mass and momentum to a differential 1-D control volume of the vessel 36,37,

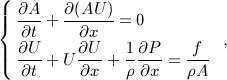
1 where *t* is the time, *x* is the distance along the vessel, *A*(*x*,*t*) is the area of the luminal cross section of the vessel, *U*(*x*,*t*) is the axial blood flow velocity averaged over the cross section, *P*(*x*,*t*) is the blood pressure averaged over the cross-section, *ρ* is the density of blood (assumed to be constant), and *f*(*x*,*t*) = − 22*πμU* is the frictional force per unit length, with *μ* = 4 mPa s the viscosity of blood.

Both models are based on data from young and healthy humans. The single-vessel model is uniform and contains a single peripheral reflection site, which simplifies our initial assessment of WIA. The 55-artery model allows us to assess WIA in the presence of tapering and multiple outflows and reflection sites; all these properties have been shown relevant for analysis of pulse wave propagation phenomena 38–41.

To account for the fluid-structure interaction of the problem and close the system of equations (1), we obtained the following explicit algebraic relationship between *P* and *A* (or *tube law*). Assuming the arterial wall to be a thin, incompressible, homogeneous, isotropic, Voigt-type visco-elastic membrane, which deforms axisymmetrically, each cross-section independently of the others, we can relate blood pressure (*P*) to luminal cross-sectional area (*A*) through 32

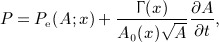
2 with

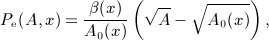
3

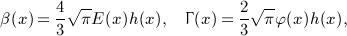


where *P*_e_ is the elastic component of pressure, *h*(*x*) is the wall thickness, *E*(*x*) is the Young's modulus, *ϕ*(*x*) is the wall viscosity, and *A*_0_(*x*) is the reference area at *P* = 0 and 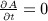. For some simulations, we considered a purely elastic tube law (i.e. *P* = *P*_*e*_) as it is assumed in WIA.

**Table II:** Parameters of the single-vessel model of the human thoracic aorta coupled to a three-element Windkessel model of the rest of the systemic circulation (Figure 3(b)).

Parameter	Value
Length, *l*	24.137 cm
Radius at diastolic pressure, *r*_d_	1.2 cm
Wall thickness, *h*	1.2 mm
Blood density, *ρ*	1,060 Kg m ^ − 3^
Young's modulus, *E*	400.0 kPa
Mean flow rate,	6.170 l min ^ − 1^
Windkessel resistance, *R*_1_	11.75 Pa s cm ^ − 3^
Windkessel compliance, *C*	10.16 mm ^3^ Pa ^ − 1^
Windkessel resistance, *R*_2_	111.67 Pa s cm ^ − 3^

The resulting wave speed at mean pressure is 5.2 m s ^ − 1^.

**Table III:** Parameters of the 55-artery model (Figure 3(e)). 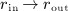, mean cross-sectional radii at the inlet and outlet of the arterial segment (radii decrease linearly); 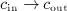, mean wave speed at the inlet and outlet of the segment.

				Mean	Mean	Peripheral	Peripheral	Wall
Arterial segment	Length	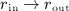	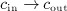	pressure	flow	resistance	compliance	viscosity
name	(cm)	(mm)	(m s ^ − 1^)	(kPa)	(ml s ^ − 1^)	(GPa s m ^ − 3^)	(m ^3^ GPa ^ − 1^)	(kPa s)
1. Ascending aorta	5.8	15.4 → 15.4	4.0 → 4.0	13.34	102.8	—	—	0.5
2. Aortic arch A	2.3	13.2 → 12.6	4.2 → 4.2	13.33	89.3	—	—	0.5
3. Brachiocephalic	3.9	10.6 → 9.4	4.5 → 4.6	13.32	13.5	—	—	1.0
4. R. subclavian	3.9	6.0 → 4.7	5.3 → 5.7	13.32	7.0	—	—	1.0
5. R. common carotid	10.8	5.7 → 2.9	5.3 → 6.5	13.29	6.5	—	—	6.0
6. R. vertebral	17.1	1.9 → 1.4	8.1 → 8.7	11.98	2.3	4.51	0.090	6.0
7. R. brachial	48.5	4.2 → 2.4	6.4 → 7.5	12.68	4.7	—	—	2.5
8. R. radial	27.0	1.9 → 1.6	8.0 → 8.4	10.82	2.4	3.96	0.099	6.0
9. R. ulnar A	7.7	1.9 → 1.7	8.0 → 8.2	12.25	2.3	—	—	6.0
10. R. interosseous	9.1	1.1 → 0.9	9.5 → 10.0	11.89	0.2	63.22	0.032	6.0
11. R. ulnar B	19.7	1.6 → 1.4	8.4 → 8.7	10.08	2.2	3.96	0.077	6.0
12. R. internal carotid	20.5	2.9 → 2.2	7.1 → 7.7	12.29	5.8	1.88	0.259	6.0
13. R. external carotid	18.7	1.3 → 0.8	9.1 → 10.4	9.53	0.7	10.42	0.193	6.0
14. Aortic arch B	4.5	11.2 → 10.9	4.4 → 4.4	13.28	83.8	—	—	0.5
15. L. common carotid	16.0	5.1 → 2.5	5.5 → 6.8	13.18	5.5	—	—	6.0
16. L. internal carotid	20.5	2.2 → 1.7	7.7 → 8.2	11.1	5.1	1.88	0.189	6.0
17. L. external carotid	18.7	1.0 → 0.6	9.8 → 11.1	7.21	0.5	10.42	0.173	6.0
18. Thoracic aorta A	6.0	10.4 → 9.9	4.5 → 4.6	13.25	76.5	—	—	0.5
19. L. subclavian	3.9	5.7 → 4.4	5.3 → 5.8	13.26	7.2	—	—	1.0
20. L. vertebral	17.0	1.9 → 1.4	8.1 → 8.7	11.98	2.3	4.51	0.090	6.0
21. L. brachial	48.5	4.2 → 2.4	6.4 → 7.5	12.64	4.9	—	—	2.5
22. L. radial	27.0	1.8 → 1.4	8.2 → 8.7	10.25	2.2	3.96	0.085	6.0
23. L. ulnar A	7.7	2.2 → 2.2	7.7 → 7.7	12.40	2.7	—	—	6.0
24. L. interosseous	9.1	0.9 → 0.9	10.0 → 10.0	11.94	0.2	63.22	0.028	6.0
25. L. ulnar B	19.7	2.1 → 1.9	7.8 → 8.0	11.54	2.6	3.96	0.130	6.0
26. Intercostals	9.2	6.6 → 4.9	5.1 → 5.6	13.20	2.0	6.00	0.104	0.5
27. Thoracic aorta B	12.0	8.6 → 6.7	4.7 → 5.1	13.05	74.5	—	—	0.5
28. Abdominal aorta A	6.1	6.3 → 6.3	5.2 → 5.2	13.00	61.3	—	—	0.5
29. Celiac A	2.3	4.1 → 3.6	5.9 → 6.1	13.05	13.2	—	—	0.5
30. Celiac B	2.3	2.7 → 2.5	6.7 → 6.8	13.04	8.9	—	—	0.5
31. Hepatic	7.6	2.8 → 2.3	7.2 → 7.6	12.97	4.3	2.72	0.205	2.5
32. Gastric	8.2	1.6 → 1.5	8.4 → 8.5	12.22	2.6	4.06	0.082	6.0
33. Splenic	7.2	2.2 → 2.0	7.7 → 7.9	12.45	6.3	1.74	0.140	6.0
34. Superior mesenteric	6.8	4.1 → 3.7	5.9 → 6.1	13.00	16.7	0.70	0.481	1.0
35. Abdominal aorta B	2.3	6.0 → 5.9	5.3 → 5.3	13.00	44.6	—	—	0.5
36. L. renal	3.7	2.7 → 2.7	6.7 → 6.7	12.77	13.4	0.85	0.231	2.5
37. Abdominal aorta C	2.3	6.1 → 6.1	5.2 → 5.2	12.98	31.2	—	—	0.5
38. R. renal	3.7	2.7 → 2.7	6.7 → 6.7	12.72	13.4	0.85	0.231	2.5
39. Abdominal aorta D	12.2	6.0 → 5.7	5.3 → 5.3	12.96	17.8	—	—	0.5
40. Inferior mesenteric	5.8	2.4 → 1.6	7.5 → 8.4	12.92	2.2	5.16	0.133	2.5
41. Abdominal aorta E	2.3	5.6 → 5.4	5.4 → 5.4	12.94	15.6	—	—	0.5
42. L. common iliac	6.8	4.1 → 3.6	5.9 → 6.1	12.92	7.8	—	—	1.0
43. R. common iliac	6.8	4.1 → 3.6	5.9 → 6.1	12.92	7.8	—	—	1.0
44. L. external iliac	16.6	3.3 → 3.1	6.3 → 6.3	12.72	5.9	—	—	2.5
45. L. internal iliac	5.8	2.1 → 2.1	7.9 → 7.9	12.78	1.9	5.96	0.137	6.0
46. L. femoral	50.9	2.7 → 1.9	7.3 → 7.9	11.32	2.9	—	—	6.0
47. L. deep femoral	14.5	2.1 → 1.9	7.9 → 8.0	12.02	3.0	3.58	0.127	6.0
48. L. posterior tibial	36.9	1.6 → 1.4	8.4 → 8.6	7.77	1.8	3.58	0.074	6.0
49. L. anterior tibial	39.8	1.3 → 1.1	8.9 → 9.1	6.08	1.1	4.19	0.051	6.0
50. R. external iliac	16.6	3.3 → 3.1	6.3 → 6.3	12.72	5.9	—	—	2.5
51. R. internal iliac	5.8	2.1 → 2.1	7.9 → 7.9	12.78	1.9	5.96	0.137	6.0
52. R. femoral	50.9	2.7 → 1.9	7.3 → 7.9	11.32	2.9	—	—	6.0
53. R. deep femoral	14.5	2.1 → 1.9	7.9 → 8.0	12.02	3.0	3.58	0.127	6.0
54. R. posterior tibial	36.9	1.6 → 1.4	8.4 → 8.6	7.77	1.8	3.58	0.074	6.0
55. R. anterior tibial	39.8	1.3 → 1.1	8.9 → 9.1	6.08	1.1	4.19	0.051	6.0

Mean pressures and flows calculated in the midpoint of the segment. The outflow pressure ( *P*_out_) is 1.33 kPa at each terminal branch and the blood density is *ρ* = 1,050 Kg m ^ − 3^. R., right; L., left.

We implemented all the boundary conditions of our simulations and solved matching conditions at bifurcations by taking into account the correct propagation of the characteristic information and neglecting energy losses and visco-elastic effects (see 1 for a detailed description).

Both models exhibit the following characteristic features of the pressure and flow waveforms that are observed *in vivo* under normal conditions. The foot of the pressure and flow waveforms in early systole propagates away from the heart (compare the simulated pressures in Figure 3(a,d) with the *in vivo* pressures in Figure 1(a,b)). The *pulse pressure* (the difference between the maximum, *systolic*, and minimum, *diastolic*, pressures) increases in the aorta with increasing distance from the heart (Figures 3(a,d) and 1(a,b)), whereas mean pressure gradually decreases§. In the ascending aorta, pressure features a ‘shoulder’ or point of inflection (Figures 3(a,d) and 1(a,b)). From the ascending aorta to the upper thoracic aorta, a small pressure peak is observed at the start of diastole, which forms the *dicrotic notch*. This vanishes in the lower thoracic region (Figures 3(d) and 1(a,b)), but it is observed in other proximal arteries such as the common carotid arteries (Figure 3(f)) 43. Moreover, the initial pressure increase becomes steeper and narrower in time in more peripheral locations (*wave steepening*) (Figures 3(a,d) and 1(a,b)), and a wide pressure peak appears in diastole from the abdominal aorta to the leg arteries 44,45 and in the brachial artery 46 (Figure 3(d,f)). With increasing distance from the heart, the aortic flow waveform (Figure 3(c,g)) becomes characterised by an increase in width, a reduction in the amount of reverse flow and a decrease in amplitude and mean value¶ 44,45. Reversed flow is absent in the suprarenal region of the aorta 29,47, renal arteries 44,48 and carotid arteries 29,49,50. There is, however, a region of reverse flow in early diastole in the infrarenal region of the aorta and leg arteries 47,50.

### 2.3 Traditional wave intensity analysis

**Figure 4:**
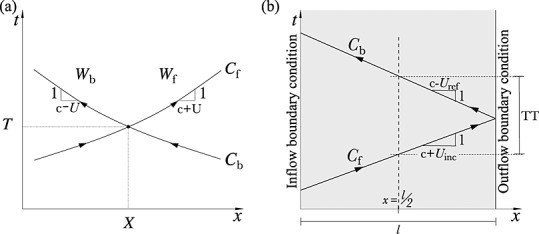
(a) In the (*x*,*t*) space, every point (*X*,*T*) of a vessel domain is intersected by a unique pair of characteristic curves 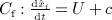 and 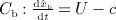 on which the respective characteristic variables *W*_f_ and *W*_b_ are invariant. (b) Sketch of our calculation of the transit time in the midpoint of the single-vessel aortic model of length *l* between an incident wavefront and its reflection. *c* is the PWV, *U* is the flow velocity and *U*_inc_ and *U*_ref_ are the average flow velocities during the propagation of the incident and reflected wavefronts, respectively.

Wave intensity analysis is derived from the system of equations (1). Velocity and pressure waveforms are decomposed into successive wavefronts, with d*P* and d*U* as changes in pressure and velocity, respectively, across a wavefront. Fluid viscous losses are assumed to be negligible locally, and *A* is assumed to depend only upon *P* through a purely elastic tube law, with uniform and constant properties. Under these conditions, Riemann's method of characteristics applied to the system of equations (1) shows (Figure 4(a)) that for any point (*X*,*T*) in the (*x*,*t*) space, there are two characteristic paths, *C*_f_ and *C*_b_, defined by 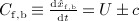, on which *W*_f_ and *W*_b_ are constant 15. The quantities *W*_f_ and *W*_b_ are generally known as the *characteristic variables* or *Riemann invariants* and satisfy

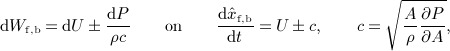
4 where the pressure-dependent *c* is the PWV, that is the speed at which pulse wavefronts travel in the absence of convective velocity (*U*) (further details are given in Appendix A). For the purely elastic tube law given by Equation (3),

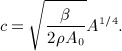
5

Under physiological flow conditions, *c* is much greater than the maximum *U*, so that *U* + *c* > 0 and *U* − *c* < 0 (i.e. the flow is subcritical). Therefore, changes in *P* and *U* propagate in the forward and backward directions (we define the forward direction as the direction of mean blood flow, in which *x* increases) with speeds of *U* + *c* and *U* − *c*, respectively.

*In vivo* measurements of *P* and *U* with time are typically taken at a fixed point *x* = *X*, rather than along a characteristic line. Solving the two equations in (4) at *x* = *X* for d*P* and d*U* yields

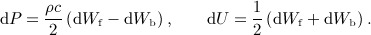
6 Wave intensity ( d*I*) is defined as 15,16

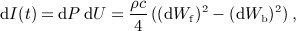
7 which is the flux of energy per unit area carried by the wavefront as it propagates and has dimensions of power/unit area and SI units W m ^ − 2^. Note that d*I* is calculated with time at a fixed point *x* = *X*. According to Equation (7), d*I* is positive if 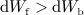 and negative if 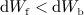. Therefore, d*I* ‘measures’ the importance with time of changes in *P* and *U* in the forward and backward directions at *x* = *X*. Whenever d*I* > 0, forward changes in *P* and *U* dominate over backward changes; the flow is accelerated if d*P* > 0 and decelerated if d*P* < 0. Whenever d*I* < 0, backward changes in *P* and *U* dominate over forward changes; the flow is accelerated if d*P* < 0 and decelerated if d*P* > 0.

#### 2.3.1 Application to measured data

Given simultaneous measurements of *P*(*t*) and *U*(*t*) at an arterial site of the numerical models or rabbit, we used a Savitzky–Golay filter to smooth d*P*(*t*) = *P*(*t* + d*t*) − *P*(*t*) and d*U*(*t*) = *U*(*t* + d*t*) − *U*(*t*), where d*t* is the time between two adjacent sampling points of *P* or *U*. This filter is commonly used for WIA because it preserves peaks in d*P* and d*U* (and hence d*I*) 16,51,52 ,p. 650. We normalised the value of d*I* given by Equation (7) by d*t*^2^ to make the magnitude of d*I* independent of the sampling frequency. We used customised matlab software (The MathWorks, Inc., MA, USA) for our data analysis.

#### 2.3.2 Forward and backward waveforms

The measured waveforms *P*(*t*) and *U*(*t*) can be separated into forward-travelling (*P*_f_(*t*), *U*_f_(*t*)) and backward-travelling (*P*_b_(*t*), *U*_b_(*t*)) components, that is 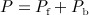 and 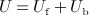. Separating d*P* and d*U* into changes across the forward ( d*P*_f_, d*U*_f_) and backward ( d*P*_b_, d*U*_b_) wavefronts, that is 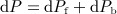 and 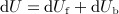, and using the *water hammer* equations,

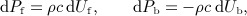
8 yield 15

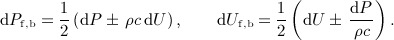
9 A derivation of Equations (8) from the system of equations (1) using the method of characteristics is given in 15. In Appendix A, we provide an alternative derivation of Equations (8) by directly applying conservation of mass and momentum to a control volume moving with the forward or backward pulse wavefronts.

If the PWV (*c*) is known, Equation (9) allows us to obtain *P*_f,b_(*t*) and *U*_f,b_(*t*) from the measured *P*(*t*) and *U*(*t*) by adding the differences d*P*_f,b_(*t*) and d*U*_f,b_(*t*), that is 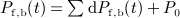 and 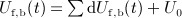. We considered the integration constants *P*_0_ and *U*_0_ to be half the pressure and velocity, respectively, at the end of diastole. Thus, wave intensity ( d*I*) at a fixed point *x* = *X* can be separated into forward ( d*I*_f_ > 0) and backward ( d*I*_b_ < 0) components,

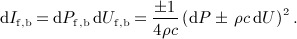
10

We can calculate *c* from simultaneous measurements of *P* and *U* using the *PU*–loop 15,53 method (see Appendix B for more details) or from two measurements of *P* (or *U*) at two different sites using foot-to-foot, least squared difference or cross-correlation techniques 25.

### 2.4 Wave reflections

At sites of impedance mismatch, pulse waves are partly reflected and partly transmitted. Using a linearised version of the 1-D equations (1), we can relate the changes in pressure and flow velocity across the reflected wavefront ( d*P*_ref_, d*U*_ref_) in the parent and two daughter vessels of an arterial bifurcation to the corresponding changes in pressure and velocity in the incident wavefront ( d*P*_inc_, d*U*_inc_) through

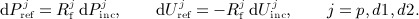
11 The superscripts *p*, *d*1 and *d*2 refer to the parent and first and second daughter vessels, respectively. The reflection coefficients for wavefronts propagating in the parent 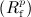 and daughter (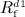 and 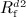) vessels can be expressed as a function of the characteristic admittance, 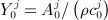, *j* = *p*,*d*1,*d*2, where *c*_0_ is the PWV at zero pressure (see Appendix C for a detailed derivation of Equations (11) and (12)),

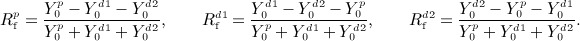
12

At the outlet of each terminal branch coupled to a single resistance (*R*_1_) we have 42

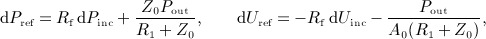
13 where *P*_out_ is the outflow pressure, *R*_f_ = (*R*_1_ − *Z*_0_)/(*R*_1_ + *Z*_0_) is the local reflection coefficient and *Z*_0_ = 1/*Y*_0_ is the characteristic impedance of the terminal branch. Note that *R*_1_ = *Z*_0_ yields *R*_f_ = 0, in which case d*P*_inc_ and d*U*_inc_ are completely absorbed by the outflow model.

From Equation (11) to Equation (13) with *P*_out_ = 0, we can relate the wave intensity of the reflected wavefront (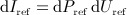) to the wave intensity of the incident wavefront (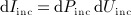) through

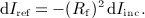
14

**Figure 5:**
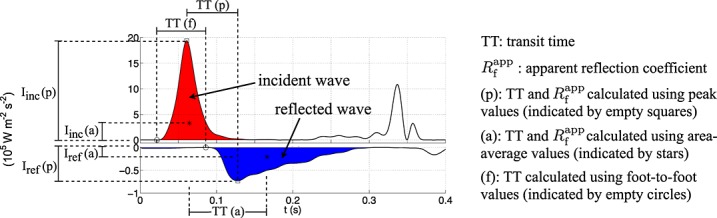
Illustration of the calculation of the transit time (TT) between the incident and reflected waves and apparent reflection coefficient 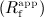 using the wave intensity profiles d*I*_f_ and d*I*_b_.

Given a wave intensity profile separated into d*I*_f_ and d*I*_b_, we defined the initial positive and negative regions as the *incident* and *reflected* waves, respectively (Figure 5). Note that these waves are made of many wavefronts across which wave intensity ( d*I*) changes. We then calculated the time of arrival (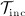 and 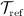) and magnitude (*I*_inc_ and *I*_ref_) of the incident and reflected waves using either peak values or area-average values; they are indicated by empty squares or stars, respectively, in Figure 5. Given the wavefronts that make up the incident, d*I*_inc_, or reflected, d*I*_ref_, wave, we defined the area-average values as

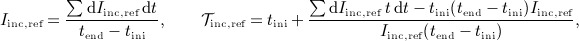
15 where the sum is taken from the start to the end of the wave, and *t*_ini_ and *t*_end_ are, respectively, the times at the start and end of the wave. These area-average values account for all the wavefronts that make up the incident and reflected waves and not only the wavefronts that define their peaks. Finally, we calculated the transit time (TT) between the incident and reflected waves and the apparent reflection coefficient (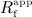) based on Equation (14) as

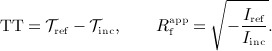
16 Thus, we obtained a pair of TT and 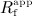 using peak values and another pair using area-average values. We also calculated TT using two foot values, which we defined as the point when d*I*_inc_ and d*I*_ref_ are greater than 1% of the peak *I*_inc_ and *I*_ref_, respectively (they are indicated by empty circles in Figure 5).

### 2.5 Haemodynamics during diastole

We can describe pressure and flow during diastole using a zero-dimensional Windkessel model. At any point in a distributed 1-D model, pressure becomes increasingly well described with increasing time in diastole by a space-independent Windkessel pressure, *p*_w_(*t*) (Figure 3(a,d,f)), given by 39,54

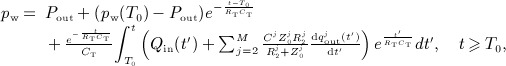
17

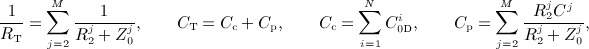
18 where *N* is the number of arterial segments, with *i* = 1 the aortic root and 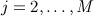 terminal segments coupled to resistance-compliance-resistance (RCR) Windkessel models (Figure 3(b)), *M* − 1 < *N* is the number of terminal branches, *Q*_in_(*t*) is the flow waveform at the aortic root, *R*_T_ is the net peripheral resistance of the arterial network, *C*_T_ is the total systemic compliance, *C*_c_ is the total conduit compliance, 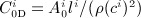 is the compliance of segment *i* (with luminal area 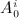, length *l*^*i*^ and PWV *c*^*i*^), *C*_p_ is the total peripheral compliance, *p*_w_(*T*_0_) is the pressure *p*_w_ at the reference time *t* = *T*_0_ (*T*_0_ = 0 in Figure 3(a,d,f)) and 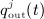 is the outflow in the terminal segment *j*. The parameters of the RCR Windkessel models are *R*_1_ = *Z*_0_, *C*, *R*_2_ and *P*_out_ (Figure 3(b)). During diastole, it is reasonable to assume *Q*_in_ = 0 and 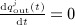, 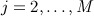, in normal conditions, which reduces *p*_w_ to

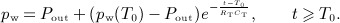
19

Equations (17) and (19) neglect nonlinearities, flow inertia and flow viscous dissipation within the 1-D model arterial segments and assume that wall compliance and fluid peripheral resistance are the dominant mechanisms of blood flow. These are reasonable assumptions towards the end of diastole, when Equation (19) provides accurate predictions of blood pressure, as shown by *in vivo* studies in dogs 55 and numerical solutions of the three-dimensional Navier–Stokes equations in compliant vessels 35.

The flow rate (*q*_w_) driven by 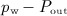 during diastole is linearly dependent on *x* in each arterial segment 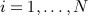 56,

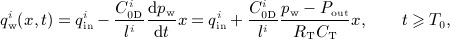
20 where 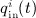 is the flow rate at the inlet of the segment. The wavefronts associated with *p*_w_ and 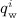 during diastole at a fixed point *x* in Segment *i*, 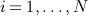, are

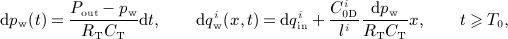
21 respectively, with d*p*_w_(*t*) uniform in space. The wave intensity 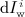 given by d*p*_w_ and 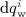 is

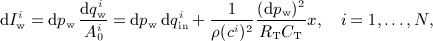
22 where 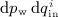 is the wave intensity at the inlet of Segment *i* and we have used 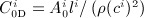.

At the ascending aorta, we have 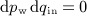, since we assumed zero flow at the root during diastole. Assuming the aorta to be a uniform vessel without branches, we have 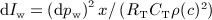 during diastole. Note that (d*p*_w_)^2^ is exponential with a time constant 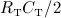 (see Equation (19)). During the systolic ejection, we can relate the velocity ( d*U*_s_) and pressure ( d*P*_s_) wavefronts using 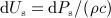 16,53, which yield an early-systole wave intensity 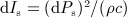. Finally, d*I*_w_ and d*I*_s_ are related through

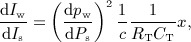
23 which shows that local (*c*) and global (*R*_T_ and *C*_T_) properties of the vasculature are responsible for the change of wave intensity from early systole to late diastole.

## 3 RESULTS AND DISCUSSION

We first study the predominant waves identified by traditional WIA in the aorta of our numerical models and rabbits and discuss their role in shaping the pressure and flow waveforms (Section 3.1 to 3.5). In particular, we show that the wave intensity profile is misleading by indicating a reflection-free period in early systole, which introduces additional error in the estimate of PWV at the aortic root by the traditional *PU*–loop method (Section 3.2). We then show that WIA does not identify peripheral reflections (Section 3.6) and assess the ability of the Windkessel model to describe haemodynamics during diastole (Section 3.7) and the modified *PU*–loop method for PWV calculation suggested in 24 (Section 3.8). We describe our new WIA (Section 3.9), which accounts for peripheral reflections using the Windkessel model, and use it to study the effect of vessel compliance and peripheral resistance on blood pressure and flow waveforms (Section 3.10). Lastly, we analyse the sensitivity of our new WIA to sampling frequency and errors in the estimate of PWV (Section 3.11).

### 3.1 Predominant wave intensity waves in the aorta

**Figure 6:**
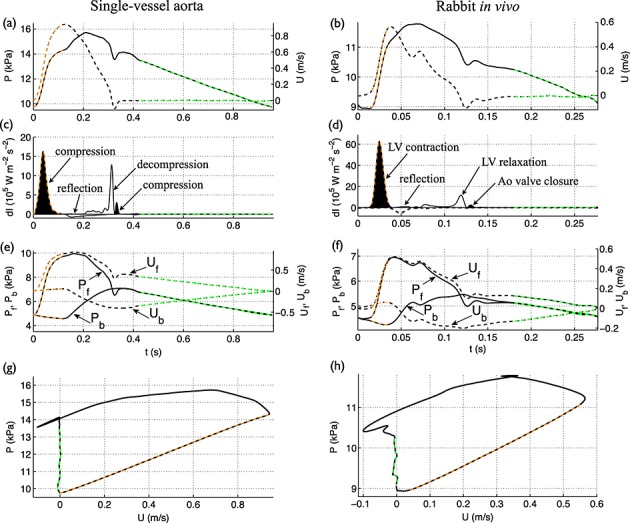
(a,b) Pressure (black solid lines) and velocity (black dashed lines) with time at the root of the (a) single-vessel aortic model and (b) *in vivo* aorta of Rabbit 8. (c,d) Forward (d*I*_f_) and backward (d*I*_b_) components of wave intensity with time (normalised by the sampling time). Shaded waves (black) accelerate blood flow, and non-shaded waves (white) decelerate blood flow. The arrows describe the type and origin of the four dominant waves in the cardiac cycle. (e,f) Forward and backward components of the pressure (*P*_f_, *P*_b_) and velocity (*U*_f_, *U*_b_) waveforms. (g,h) Pressure versus velocity (*PU*–loop). d*I*_f,b_, *P*_f,b_ and *U*_f,b_ were calculated using the PWV given by (c,e) Equation (5) with *A* the mean area or (d,f) the foot-to-foot method as described in Section 2.1. For all data contours, the apparently reflection-free period in early systole and the decay in pressure with approximately constant blood velocity are highlighted in orange and green, respectively.

At any point in the single-vessel aortic model coupled to a matched three-element Windkessel outlet (Figure 3(b)), we identified four predominant waves in the cardiac cycle from the forward ( d*I*_f_) and backward ( d*I*_b_) wave intensity profiles as follows (Figure 6(c) shows the profiles at the inlet): (i) an initial forward compression wave (i.e. travelling towards the outlet, because d*I* > 0, and with d*P* > 0) produced by the increase in blood flow at the inlet (inflow) in early systole; (ii) a backward compression wave indicating reflection of the initial wave at the outlet; (iii) a forward decompression ( d*P* < 0) wave (a ‘suction’ wave) caused by the decrease in inflow towards the end of systole; and (iv) a forward compression wave due to the short increase in inflow at the end of systole. The flow is accelerated by the first and fourth waves and decelerated by the second and third. These four waves were also observed in the ascending aorta of the 55-artery model (Figure 3(e), results not shown).

Furthermore, the four waves were observed in the ascending aorta of the rabbit (Figure 6(d)). Their timing and direction of propagation suggest that the first wave is caused by the contraction of the LV, the second is due to reflections of the initial contraction wave at downstream sites of impedance mismatch, the third is produced by the relaxation of the LV and the fourth results from the closure of the aortic valve. The first three waves have been extensively reported for the human aorta 15,16. The fourth wave has been observed in human coronary arteries 12,14; the aorta of the dog 53,57, sheep 58 and swine 59; and large arteries of the fetal lamb 20.

### 3.2 Left ventricular-contraction wave – an apparent reflection-free period in early systole

Both numerical and *in vivo* results in Figure 6(c,d) show that there is a period in early systole during which d*I*_b_ is almost zero. This indicates that changes in pressure and velocity are generated by the contraction of the LV only and not affected by wavefronts reflected in the vasculature. Indeed, the increase in pressure (Figure 6(a,b), orange dashed lines) is made up entirely of forward pressure wavefronts ( d*P*_f_) during this apparently reflection-free period (Figure 6(e,f), orange dashed lines). According to Equation (10), if d*I*_b_ = 0 in early systole, then d*P* = *ρc* d*U*; that is pressure (*P*) and flow velocity (*U*) are proportional and make the linear part of the *PU*–loop (Figure 6(g,h), orange dashed lines).

However, Figure 6(e,f) shows clearly that the backward pressure (*P*_b_) and velocity (*U*_b_) waveforms are not constant during the apparently reflection-free period in early systole: *P*_b_ is made up of backward pressure wavefronts ( d*P*_b_) that decrease *P*, and *U*_b_ is made up of backward velocity wavefronts ( d*U*_b_) that increase *U*. According to Equation (9), non-zero d*P*_b_ and d*U*_b_ indicate that d*P* = *ρc* d*U* is not satisfied. Indeed, from the slope of the *PU*–loops (Equation (B.1)) in Figure 6(g,h) we have *c*_PU_ = 4.7 m s ^ − 1^ in the aortic model and *c*_PU_ = 4.0 m s ^ − 1^ in the rabbit, which differ respectively from *c* = 5.2 m s ^ − 1^ given by Equation (5) (with *A* the mean area) and *c*_ff_ = 4.4 m s ^ − 1^ calculated using the foot-to-foot method. At the aortic root of the 55-artery model, *c*_PU_ = 3.4 m s ^ − 1^, which differs from *c* = 4.0 m s ^ − 1^ using Equation (5) with mean area. The mean error of *c*_PU_ in our ten rabbits relative to *c*_ff_ is 9.5% (Table I), which is similar to the error reported in 24 using numerical data only. Therefore, the wave intensity profile is misleading by indicating a reflection-free period in early systole, which leads to an error in the estimate of *c* by the *PU*–loop method.

### 3.3 Reflection of the left ventricular-contraction wave – pressure augmentation

Reflections of the forward wavefronts that make up the LV-contraction (compression) wave yield a reflected wave that is made up of negative velocity wavefronts ( d*U*_ref_ < 0) and positive pressure wavefronts ( d*P*_ref_ > 0), the wave labelled ‘reflection’ in Figure 6(c,d). This wave produces a *U*_b_ that decelerates the net forward flow and a *P*_b_ that augments pressure (Figure 6(e,f)), thereby generating the ‘shoulder’ or point of inflexion (Figure 6(a,b)) that defines the pressure *augmentation index* 60.

In the single-vessel aortic model, reflected wavefronts originate from the outlet, which is coupled to a matched three-element Windkessel model (Figure 3(b)). In the more realistic 1-D distributed model, however, the origin of the reflected wave is more complex. Several studies 38–41 have identified multiple reflection sites that reflect wavefronts towards the aorta, where they arrive at different times with a magnitude that decreases exponentially with time 39. The net effect of multiple reflection sites on the TT and 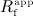 given by Equation (16) along the aorta is an apparent reflection site that appears to move away as the measurement location approaches it 40.

### 3.4 Left ventricular-relaxation wave – flow deceleration

The LV-relaxation (forward) wave drops pressure and flow during the deceleration phase towards the end of systole (Figure 6(a,b)). Flow velocity is reduced by both forward (*U*_f_) and backward (*U*_b_) velocities and becomes negative at the end of systole, whereas pressure (*P*) is reduced by only the forward pressure (*P*_f_); *P*_b_ continues increasing *P* (Figure 6(e,f)). Thus, during the LV-relaxation phase, wavefronts originating at the aortic root decrease both pressure and velocity, whereas wavefronts reflected in peripheral locations increase pressure and decelerate the flow.

Two mechanisms have been suggested to describe the origin of the LV-relaxation wave, active myocardial relaxation and flow inertia, which are discussed in 61 ,p. 95.

### 3.5 Valve wave – dicrotic notch

**Figure 7:**
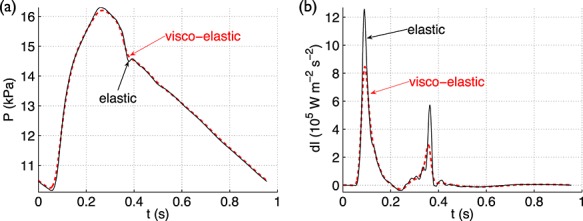
(a) Pressure and (b) wave intensity with time in the thoracic aorta of the 55-artery model (midpoint of Segment 27 in Figure 3(e)) using the visco-elastic (Equation (2)) or purely-elastic (Γ = 0) tube law.

The closure of the aortic valve produces a forward compression wave that augments pressure, creating the dicrotic notch, and accelerates the flow, from negative to zero at the root (Figure 6(a,b,c,d)). The dicrotic notch is shaped by the forward pressure component (*P*_f_) (Figure 6(e,f)), that is by wavefronts travelling from the aortic root, and progressively vanishes in the aorta with increasing distance from the heart (Figure 1(a,b)). In the thoracic aorta of the 55-artery model (Figure 3(e)), the dicrotic notch is absent if wall viscosity is modelled and is present if wall viscosity is neglected (Figure 7(a)). Therefore, wall viscosity dissipates the dicrotic notch. Wall viscosity also dissipates all predominant waves in the wave intensity profile as they travel towards distal locations, almost abolishing the valve wave (Figure 7(b)). *In vivo* measurements in humans show that the valve wave is small or absent in the brachial, radial and femoral arteries 7,11.

### 3.6 Why does wave intensity vanish during diastole? The importance of peripheral wave reflections from previous cardiac cycles

Numerical and *in vivo* wave intensity profiles in the aorta during diastole do not show any reflection of the four predominant waves discussed previously (Figure 6(c,d)). Here, we show that WIA fails to identify the important contribution to the pressure waveform of peripheral reflections, using our single-vessel (Section 3.6.1) and distributed (Section 3.6.2) models.

#### 3.6.1 Single-vessel aortic model

**Figure 8:**
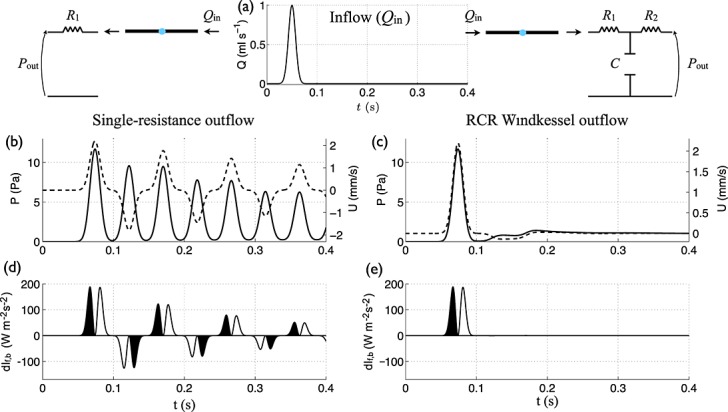
(b,c) Pressure (solid lines) and velocity (dashed lines) with time in the midpoint of the single-vessel aortic model coupled to an outflow made of a (b) single-resistance or (c) RCR Windkessel (with *R*_1_ = *Z*_0_ to minimise wave reflections 42) model. In both models, *P*_out_ = 0. The inflow (*Q*_in_) is a Gaussian-shaped wave with a peak flow rate of 1 ml s ^ − 1^ (a). (d,e) Forward (d*I*_f_) and backward (d*I*_b_) components of wave intensity with time (normalised by the sampling time). Shaded waves (black) accelerate blood flow and non-shaded waves (white) decelerate blood flow.

According to this model, wave intensity vanishes during diastole because reflected wavefronts are spread in time by peripheral compliance. To illustrate this, at the inlet of the vessel we prescribed a narrow Gaussian-shaped flow (Figure 8(a)) that approximates a compression wavefront immediately followed by a decompression wavefront. At the outlet, we first coupled a single-resistance with a zero outflow pressure, so that peripheral compliance is absent. This model generates multiple Gaussian-shaped pulse waves (Figure 8(b)). As predicted by Equation (13), both outlet and inlet change the direction of the blood flow; the outlet yields reflected waves travelling towards the inlet with 83% (*R*_f_ = (*R*_1_ − *Z*_0_)/(*R*_1_ + *Z*_0_) = 0.83) of the pressure and flow amplitudes of the incident wave, and the inlet behaves as a closed end (*R*_f_ = 1, because *Q*_in_ = 0 by the time the first pulse wave is reflected) producing reflected waves with the same amplitude as the incident wave.

The wave intensity profile is able to identify the timing, direction and magnitude of the multiple waves reflected in the model (Figure 8(d)). Equation (16) applied to the wave intensity profile in the midpoint yields errors in the 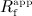 estimates smaller than 2% using peak values and 4% using area-average values (Equation (15)), both relative to the theoretical *R*_f_ = 0.83. Furthermore, the errors in the estimates of TT in this model are smaller than 1% using foot, peak and area-average values, which shows the ability of Equation (16) to estimate accurately TT and *R*_f_ in the presence of a single peripheral reflection site without peripheral compliance.

Adding peripheral compliance using, for example, a matched RCR Windkessel outflow model (i.e. with *R*_1_ = *Z*_0_ to minimise wave reflections 42, Figure 3(b)) has the effect of smoothing reflected waves (Figure 8(c)), which is necessary for obtaining physiological-like waveforms (e.g. those in Figure 6(a,c)). Although reflected waves alter the pressure and flow waveforms after *t* = 0.1 s (Figure 8(c)), the wave intensity profile does not reveal them (Figure 8(e)), because the initial pulse wave has a wave energy several orders of magnitude greater than its reflections.

#### 3.6.2 Distributed 1-D model

We can study the role of peripheral reflections in shaping the pressure waveform using the following methodology. Neglecting nonlinear effects, we can separate the pressure and flow waveforms at an arbitrary point in a given distributed model into a *conduit (or arterial) waveform*, which is made up of pulse wavefronts propagating from the aortic root and being reflected at the arterial junctions, aortic root and tapered vessels, and a *peripheral waveform*, which is made up of wavefronts originating from reflections at terminal branches. As detailed in 39, the conduit waveform is obtained by running the simulation with each terminal branch coupled to a single resistance equal to the characteristic impedance of the branch, so that any wavefront leaving the vessel is completely absorbed by the boundary condition; that is 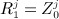 so that 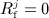 in Equation (13), 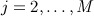. The peripheral waveform is the difference between the total and conduit waveforms.

**Figure 9:**
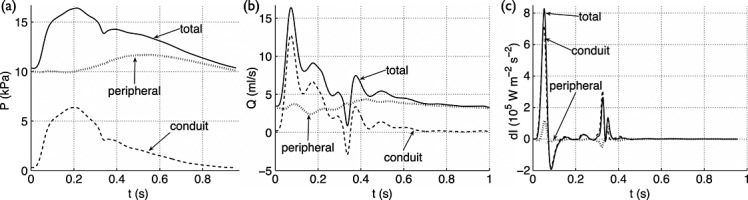
Total, conduit and peripheral (a) pressures, (b) flows and (c) wave intensities with time in the midpoint of the left common carotid artery of the visco-elastic 55-artery model (Segment 15 in Figure 3(e)).

In the aorta and main branches, most of the pressure waveform at the start of systolic ejection is made up of peripheral reflections, as Figure 9(a) shows for the left common carotid artery. These reflections originated in previous cardiac cycles, because they are present before the start of cardiac ejection in the current cardiac cycle. The maximum contribution of peripheral reflections to the pressure waveform occurs in early diastole, when these reflections produce a concave shape in the proximal arteries of our 55-artery model (Figures 3(d) and 9(a)). This feature of the pressure waveform is sometimes present *in vivo* in the aorta of the human 3,60,62, rabbit (e.g. Figure 6(b)) and dog 5,55 and in the human carotid, brachial and radial arteries 6–9,11. Furthermore, in the carotid artery, peripheral reflections provide a pressure gradient that drives less than 50% of the mean blood flow in systole, but more than 90% of the mean flow in diastole (Figure 9(b)). All wavefronts that did not originate as peripheral reflections and which make up the conduit waveform are responsible for the features of the pressure and flow waveforms in systole (e.g. pulse pressure and dicrotic notch, Figure 9(a); flow amplitude, Figure 9(b)). In diastole, these wavefronts produce a conduit waveform that decreases exponentially and contributes little to the pressure and flow waveforms in the next cardiac cycle.

Although the diastolic pressure and flow waveforms in our distributed 1-D model are mainly made up of peripheral reflections, WIA fails to identify them. The wave intensity profile only reveals the waves that shape the conduit waveform, as Figure 9(c) shows: the conduit pressure and flow waveforms produce a profile similar in shape and magnitude to the ‘total’ profile, whereas the ‘peripheral’ wave intensity profile has a considerably smaller magnitude than the ‘total’ profile, because changes in pressure and velocity across the wavefronts that make the peripheral waveform are smaller than those that make the conduit waveform. As a result, the apparent reflection coefficient given by Equation (16) accounts little for peripheral reflections. Indeed, calculation of reflection coefficients using forward and backward pressures produces smaller errors than using wave intensity profiles 18.

We can use Equation (23) to quantify the reduction in wave intensity in the aorta from systole ( d*I*_s_) to diastole ( d*I*_w_) because of net peripheral resistance (*R*_T_) and total compliance (*C*_T_) acting on the blood flow through the Windkessel effect. Taking *x* = 20 cm, *c* = 5 m s ^ − 1^ and 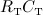 = 1.8 s as representative values for the thoracic aorta and 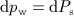, then d*I*_w_ is 2% of d*I*_s_. However, 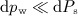 because the magnitude of pulse wavefronts decreases as they get reflected and attenuated in the arterial network. Therefore, d*I*_w_ is several orders of magnitude smaller than d*I*_s_ (Figure 7(b)).

Next, we discuss the validity of the Windkessel model to study haemodynamics during diastole.

### 3.7 Using the Windkessel model to study haemodynamics during diastole

Our rabbit *in vivo* data show that the space-independent pressure *p*_w_(*t*) given by Equation (19) is able to describe pressure towards the end of diastole. Fitting Equation (19) to the decline in pressure during asystole measured along the aorta and iliac artery at 5 cm increments (at least five measurements were taken for each rabbit) produced a small variability of the time constant (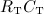) and asymptotic pressure (*P*_out_); for example, 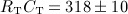 ms and *P*_out_ = 4.5 ± 0.2 kPa in rabbit 8 (Figure 2(c,d) and Table I).

Despite the rabbit having about four times the human heart rate, human and rabbit pressure waveforms are similar in shape when normalised in time (compare Figures 1(a,b)). Therefore, pressure in diastole falls at a faster rate with time in the rabbit, as indicated by the average time constant 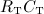 in the rabbit ( 0.29 ± 0.02 s; Table I) being one order of magnitude smaller than in the human ( 1.79 ± 0.13 s 46). This is due to a smaller total systemic compliance (*C*_T_) in the rabbit ( 0.21 ± 0.05 mm ^3^ Pa ^ − 1^) than in the human ( 17.0 ± 1.0 mm ^3^ Pa ^ − 1^ 46); vessels are less compliant (i.e. *C*_0D_ = *A*_0_*l*/(*ρc*^2^) is smaller) for the shorter and thinner rabbit vessels, despite having similar PWV to the human vessels.

In agreement with Equation (20), the flow in the last part of diastole increases linearly from the inlet to the outlet of the single-vessel aortic model (Figure 3(c)) and from the aortic root to the thoracic aorta of the 55-artery model (Figure 3(g)). For a given point *x* in the aorta, the flow towards the end of diastole decays exponentially (Figure 3(c,g,h)). Furthermore, the *PU*–loop is approximately linear in the last part of diastole (Figure 6(g,h), green dashed lines), which is in agreement with *p*_w_ and 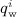 being proportional in the last part of diastole, according to Equation (20).

These results provide further evidence to support the conclusions in 35,55 that the Windkessel model has application for study of haemodynamics in diastole. Application of this model enables us to account for wave reflections originating from previous cardiac cycles and thereby modify the *PU*–loop method (Section 3.8) and WIA (Section 3.9).

### 3.8 Modified 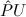
–loop method

**Figure 10:**
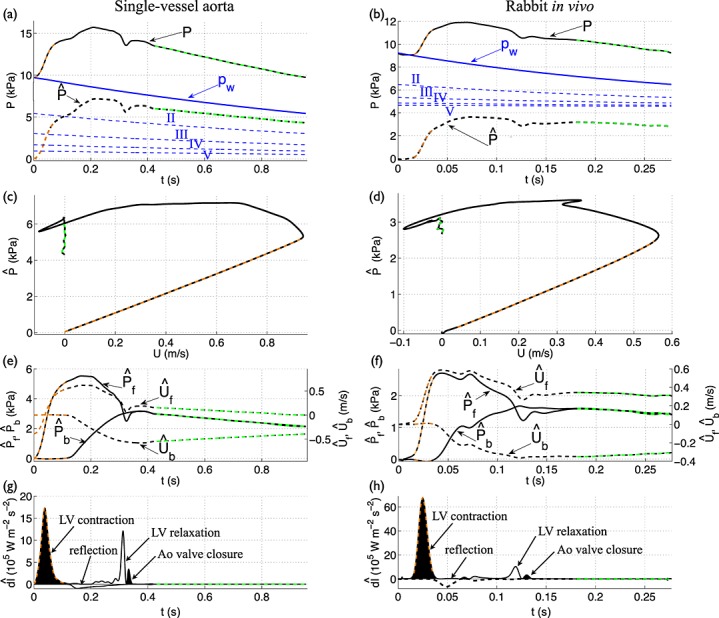
(a,b) Pressure (*P*) (black solid lines), Windkessel pressure decay (*p*_w_) prolonged from the previous cardiac cycle (blue lines) and 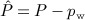 (black dashed lines) with time at the root of the (a) single-vessel aortic model and (b) *in vivo* aorta of Rabbit 8. Contributions to *P* from the second (II), third (III), fourth (IV) and fifth (V) previous cycles are shown in blue dashed lines. Modified (c,d) 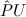
–loop, (e,f) forward and backward components of the pressure 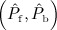 and velocity 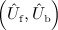 waveforms, and (g,h) forward 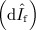 and backward 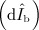 components of wave intensity with time (normalised by the sampling time). The arrows describe the origin of the four dominant waves in the cardiac cycle. 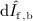, 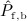 and 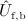 were calculated using the PWV given by (e,g) Equation (5) with *A* the mean area or (f,h) the foot-to-foot method as described in Section 2.1. For all data contours, the reflection-free period in early systole and the decay in pressure with approximately constant velocity are highlighted in orange and green, respectively.

Figure 10(c,d) shows pressure cleared of contributions of waves reflected in previous cardiac cycles 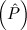 versus flow velocity (*U*) at the root of the single-vessel aortic model and *in vivo* rabbit aorta. In the numerical model, these contributions were obtained by prolonging the decaying *p*_w_ given by Equation (19) from the previous cardiac cycle into the current cycle, with the time constant (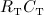) and asymptotic pressure (*P*_out_) given by the theoretical parameters of the model. In the rabbit, 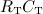 and *P*_out_ were calculated by fitting an exponential function of the form given by Equation (19) to the decline in pressure during several asystole generated along the aorta and iliac artery at 5 cm increments (Figure 2(b,e)). The values of 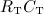 and *P*_out_ were taken to be the corresponding mean values for each rabbit (Figure 2(c,d)). Figure 10(a,b) shows *p*_w_ prolonged from the previous cardiac cycle and the pressure waveform 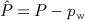, which is cleared of contributions of reflected waves originating from previous cardiac cycles, for the numerical and *in vivo* data. We did not clear *U* of contributions of reflected waves originating from previous cycles, because *U* approximates zero at the aortic root. In more distal locations, these contributions could be obtained by prolonging the decaying *U* from the previous cardiac cycle into the current cycle. Theoretically, the decaying *U* should be approximated by 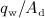 given by Equation (20), with *A*_d_ the diastolic area.

The backward pressure 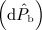 and velocity 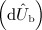 wavefronts calculated by applying Equation (9) to 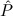 and *U* are zero in early systole (i.e. 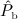 and 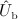 are constant, as shown in Figure 10(e,f), orange dashed lines) and, hence, 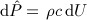 is satisfied in early systole (unlike d*P* = *ρc* d*U*). As a result, the slope of 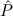 versus *U* in Figure 10(c,d) (orange dashed lines) yields 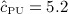 m s ^ − 1^ in the aortic model, in agreement with the theoretical value calculated using Equation (5) with *A* the mean area, and 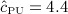 m s ^ − 1^ in the rabbit aorta, in agreement with the value calculated using the foot-to-foot method (Table I). In all ten rabbits, the modified 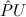
–loop method provided smaller errors than the traditional *PU*–loop method in the estimates of PWV in the ascending aorta, relative to the foot-to-foot estimates (three last columns of Table I). The mean relative errors for the ten rabbits were 1.3% using the modified 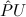
–loop method and 9.5% using the traditional *PU*–loop method, which are comparable to the errors reported in 24 obtained from numerical data.

Our results support the use of the modified 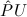
–loop method when an exponential fit to the decline in pressure during diastole is possible. An exponential fit may be challenging when using *in vivo* data without asystole and when the pressure decay does not develop into an exponential (e.g. see Figure 6(b)) 63. Tapering and energy losses are other potential sources of error when using the *PU*–loop, because they are not accounted for in the derivation of Equation (B.1) (see Appendices A and B).

Lastly, we note that it is not necessary to eliminate the pressure contribution from previous cardiac cycles in order to estimate accurately the local PWV from simultaneous *P* and luminal area (*A*) measurements at an arbitrary location in the arterial network. This is because pressure and luminal area wavefronts are reflected with the same reflection coefficient (*R*_f_), whereas velocity wavefronts are reflected with − *R*_f_ and, hence, reflections from previous cardiac cycles have a similar effect on *P* and *A*, unlike on *P* and *U* (see Appendix C). Applying Equation (B.3) from Appendix B to *P* and *A* at any point of the visco-elastic 55-artery model (Figure 3(e)) yields errors in the estimates of PWV smaller than 2%, relative to the theoretical PWV obtained using Equation (6) with *A* the mean area over the cardiac cycle. Such a small error, however, remains to be verified using *in vivo* *P* and *A* data.

### 3.9 Novel wave intensity analysis

We propose a modified WIA that consists of (i) the new wave intensity 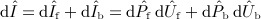 calculated using 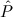 and *U* to study haemodynamics during systole (Section 3.9.1); (ii) the Windkessel model given by Equation (19) to study haemodynamics during diastole (as we showed in Section 3.7) and quantify the contribution to the pressure waveform of wave reflections originating from previous cardiac cycles (Section 3.9.2); and (iii) the Windkessel model given by Equation (17) (with 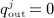, 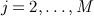, when analysing *in vivo* data) to analyse the effect on the pressure waveform during both systole and diastole of vessel compliances, peripheral resistances, outflow pressures and the flow at the root (*Q*_in_(*t*)) (Section 3.10).

#### 3.9.1 Haemodynamics during systole

**Figure 11:**
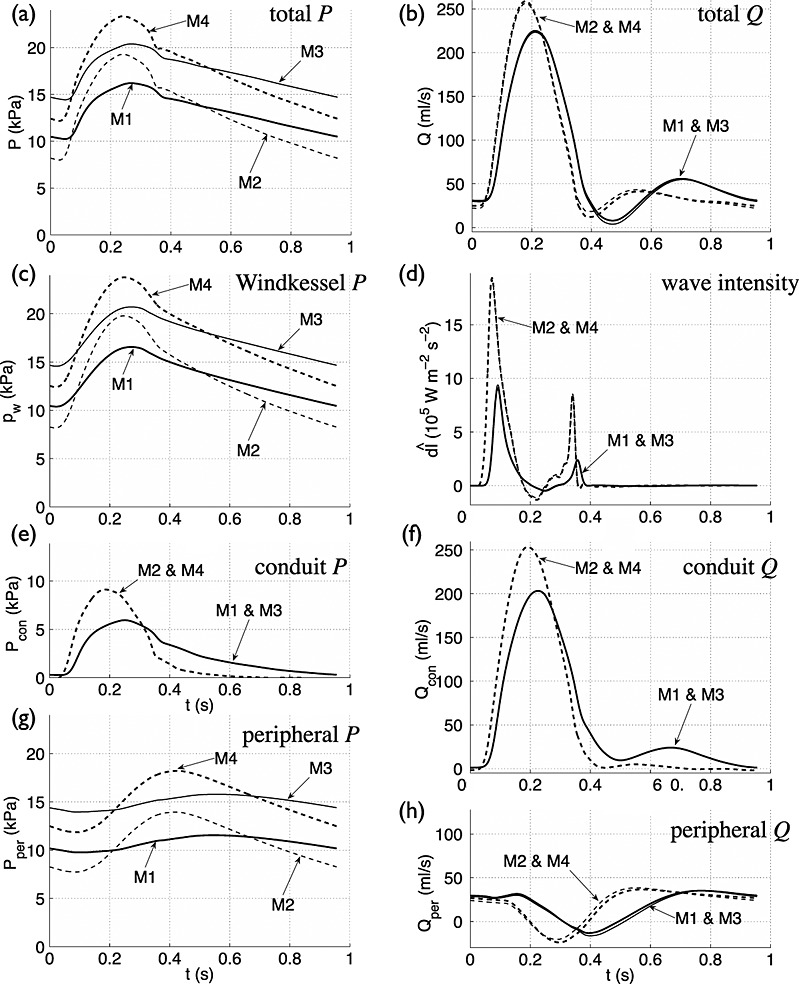
(left) Total (a), Windkessel (c), conduit (e) and peripheral (g) pressures with time in the thoracic aorta (midpoint of Segment 27 in Figure 3(e)) of the *normal young* (M1), *normal old* (M2), *hypertensive young* (M3) and *hypertensive old* (M4) models described in Section 3.10. (right) Total (b), conduit (f) and peripheral (h) flow rates and modified wave intensity (d) with time at the same location and models.

The modified 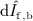 profiles are not influenced by wave reflections originating from previous cardiac cycles and, hence, allow us to do WIA starting from a ‘true’ reflection-free period in early systole. In normal conditions, they are very similar to the traditional d*I*_f,b_ profiles (e.g. compare Figure 6(c,d) with Figure 10(g,h), and ‘visco-elastic’ in Figure 7(b) with ‘M1’ in Figure 11(d)) and contain the four predominant waves described in Sections 3.1 – 3.5. However, using foot values of 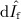 and 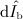 decreases the error of the TT estimate provided by Equation (16) in the single-vessel aortic model coupled to a matched RCR Windkessel model (Figure 3(b)). From the profiles d*I*_f_ and d*I*_b_ in the midpoint of the vessel, calculated with *c* at diastolic pressure, we obtained TT = 67 ms using peak values, TT = 102 ms using area-average values (Equation (15)) and TT = 65 ms using foot values. Their errors are, respectively, 31%, 100% and 27%, relative to the theoretical 51 ms calculated as 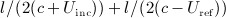, according to the characteristics analysis (see Figure 4(b)), where *l* = 24.1 cm is the vessel length, *c* = 5.0 m s ^ − 1^ is the theoretical PWV at diastolic pressure given by Equation (5), *U*_inc_ = 0.04 m s ^ − 1^ is the average flow velocity during the propagation towards the outlet of the wavefront that forms the foot of the incident wave, and *U*_ref_ = 0.5 m s ^ − 1^ is the average flow velocity during the propagation towards the inlet of the wavefront that forms the foot of the reflected wave. Similar relative errors were obtained at any point in the vessel, suggesting that foot values should be used for the calculation of TT. The relative error given by foot values of 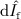 and 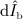 was reduced to 10%.

Conceptually, the new wave intensity profile should be used to study haemodynamics during systole. In practice, however, the new and traditional wave intensity profiles may be very similar due to measurement errors.

#### 3.9.2 Contributions of wave reflections from previous cardiac cycles

The contribution of wave reflections from several previously occurring cardiac cycles to the pressure waveform (*P*) can be studied by prolonging the decay of *p*_w_ given by Equation (19) at the end of each previous cycle. Figure 10(a,b) shows the contribution to *P* at the aortic root in the numerical model and rabbit *in vivo* of the second (II), third (III), fourth (IV) and fifth (V) previous cycles, and Table IV quantifies them. In the rabbit, the pressure at which flow to the microcirculation ceases (*P*_out_) contributes 4.5 kPa to *P*. In the numerical model, *P*_out_ was set to zero and does not play a role in shaping *P*. In both the numerical model and the rabbit, the current cycle contributes to *P* more than any previous cycle, providing the systolic features of pressure that can be studied using 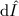, and, in the rabbit, the diastolic concave shape. Pressure contributions from previous cycles decrease exponentially, with earlier cycles generating a smaller percentage of *P* than later cycles.

**Table IV:** Contribution from the current cardiac cycle, the first (I), second (II), third (III), fourth (IV) and fifth (V) previous cycles, and the outflow pressure (*P*_out_) to the pressure waveform (*P*) at the aortic root of the single-vessel aortic model (Figure 10(a)) and *in vivo* Rabbit 8 (Figure 10(b)).

Cardiac cycle	Single-vessel aorta	Rabbit *in vivo*
Current	41.9	27.0
I	25.7	17.4
II	14.3	7.3
III	8.4	3.0
IV	4.0	1.3
V	2.5	0.5
*P*_out_	0	43.0

Each contribution is quantified using *p*_w_, as described in Section 3.9.2, and is expressed in % relative to the area under *P*.

Because conduit pressures vanish almost completely by the end of diastole (Figure 9(a)), pressure contributions from previous cardiac cycles are mostly made up of reflected waves originating from peripheral reflection sites. These reflected waves persist for several cardiac cycles, because they become trapped within the arterial network between the aortic valve and peripheral vessels 39. In diastole, the valve is closed and so reflects backward wavefronts with a reflection coefficient close to one 39,56. As a result, forward and backward pressures (*P*_f,b_ and 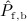) are equal and forward and backward velocities (*U*_f,b_ and 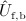) are opposite, as Figures 6(e,f) and 10(e,f) show.

### 3.10 Effect of vessel compliance and peripheral resistance

We use our modified WIA to study the effects of changes in vessel compliance and peripheral resistance on the pressure and flow waveforms in the thoracic aorta of the visco-elastic 55-artery model (Figure 3(e)), with the parameter values described in Table III (hereinafter referred to as *Model M1, normal young*) and the following three variations:

- *Model M2, normal old*: The elastic modulus *E* is increased three-fold in all the arterial segments (except for the terminal branches) to decrease the total conduit compliance (*C*_c_) and model the increase in the stiffness of the arterial wall with ageing 64.
- *Model M3, hypertensive young*: The total resistance *R*_1_ + *R*_2_ at the outflow of each 1-D model terminal branch (Figure 3(b)) is increased by 40% to raise blood pressure.
- *Model M4, hypertensive old*: The changes introduced in M2 and M3 are combined.

Using the Windkessel pressure (*p*_w_) as a zero-order approximation to the pressure waveform (*P*) provides relevant information on the effects of vessel compliance and resistance. First, we note that the total compliance (*C*_T_) and peripheral resistance (*R*_T_) appear in the term 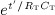 of the integrand of Equation (17). This term increases the contribution to *P* of the decaying flow out of the heart (*Q*_in_; Figure 3(g), ‘Root’) in the last part of systole; the greater *C*_T_ and *R*_T_ are, the more they spread the shape of *Q*_in_ (and hence *p*_w_) in time. Furthermore, the magnitude of *P* produced by *Q*_in_ in systole decreases with increasing *C*_T_ through the term 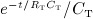 multiplying the integral. Thus, the pulse pressure decreases with increasing *C*_T_. According to Equation (18), *C*_T_ increases with the increasing compliance of each segment in the arterial network (*C*_0D_), which is greater in *young* models (M1 and M3) than in *old* models (M2 and M4). As a result, the pulse pressure of both *p*_w_ and *P* at the thoracic aorta is less in *young* than *old* models (Figure 1(a,c)). *In vivo* measurements in humans show an increase in pulse pressure with age 62 and disease (such as atherosclerosis and diabetes) 65, which can be explained by a decrease in vessel compliance. Lastly, both *P* and *p*_w_ decay in diastole at a greater rate in *old* than in *young* models (Figure 1(a,c)), because the time constant (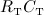) is less in *old* than *young* models, in agreement with Equation (19).

Using *p*_w_ to quantify the portion of *P* at the thoracic aorta originating from previous cardiac cycles reveals similar contributions to *P* from the first previous cycle in the four models studied (Table V). However, in *old* models a smaller portion of *P* is made up of reflected waves originating from earlier cycles because *p*_w_ falls at a faster rate with decreasing *C*_T_, and in *hypertensive* models a greater portion of *P* comes from earlier cycles (compare M1 with M3 and M2 with M4 in Table V) because *p*_w_ falls at a slower rate with increasing *R*_T_.

**Table V:** Contribution from the current cardiac cycle, the first (I), second (II), third (III), fourth (IV) and fifth (V) previous cycles, and the outflow pressure (*P*_out_) to the pressure waveform in the thoracic aorta (midpoint of Segment 27 in Figure 3(e)) of the *normal young* (M1), *normal old* (M2), *hypertensive young* (M3) and *hypertensive old* (M4) models.

Cardiac cycle	M1	M2	M3	M4
Current	38.6	58.9	30.8	48.7
I	24.6	21.4	23.2	24.5
II	12.8	6.6	14.5	10.7
III	6.7	2.0	9.0	4.7
IV	3.5	0.6	5.6	2.1
V	1.8	0.2	3.5	0.9
*P*_out_	10.2	10.2	7.7	7.7

We can study how *C*_T_ and *R*_T_ affect systolic haemodynamics using the wave intensity profile 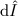. The peak values of 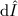 are greater with decreasing *C*_T_ and, hence, the energy carried by pulse wavefronts in *old* models (M2 and M4) is greater than in *young* models (M1 and M3) (Figure 11(d)). This result suggests that the LV must produce more energy to propel the same amount of blood flow throughout the vasculature of *old* models. On the other hand, *normal* and *hypertensive* models of the same age (i.e. M1 and M3 or M2 and M4) have similar contours of 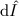; that is, changes in *R*_T_ have little effect on 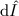. Therefore, peripheral reflections have a minor effect on the flux of energy in the thoracic aorta in systole. This result provides further evidence to support our result in Section 3.6 on the inability of the wave intensity profile to identify peripheral reflections. Furthermore, this result suggests that changes in *R*_T_ have little effect on aortic augmentation index, in agreement with 41.

The foot of the incident wave in the 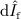 profile occurs earlier in *old* models (Figure 11(d)), which feature greater PWV in proximal vessels, but the TT between the incident and reflected waves calculated using Equation (16) with foot values changes by less than 2 ms in all four models. Furthermore, the apparent reflection coefficient 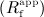 is not affected by changes in *R*_T_, but it increases with decreasing *C*_T_; 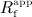 is 7% greater in M2 than in M1. Different results are obtained, however, in the single-vessel aortic model; TT decreases with decreasing *C*_T_ and increasing *R*_T_, in agreement with theoretical predictions, and 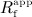 increases with increasing *C*_T_ and *R*_T_ (see supplementary material for further details). Therefore, varying *C*_T_ and *R*_T_ has a different effect on TT and 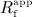 in the presence of one or multiple peripheral reflection sites.

We can further investigate the effects of *C*_T_ and *R*_T_ on the pulse waveform by calculating conduit and peripheral waveforms as described in Section 3.6.2. Decreasing *C*_T_ increases the amplitude of both the conduit and peripheral pressure waveforms, but increasing *R*_T_ only increases the magnitude (and not the shape) of the peripheral waveform (Figure 1(e,g)). These results indicate that vessel compliance has a similar effect on reflections originating from internal junctions, aortic root and tapered vessels to those originating from the periphery, whereas peripheral resistances only affect reflections originating from the periphery. Thus, ‘conduit’ and ‘peripheral’ mechanisms underlie changes in pulse pressure, whereas only ‘peripheral’ mechanisms underlie changes in mean pressure. Changes in *C*_T_ affect both the conduit and peripheral flow waveforms (Figure 1(f,h)). In particular, the amplitude of the conduit waveform increases in *old* models, which leads to greater peak flows and less flow damping (Figure 1(b)). Changes in *R*_T_ have little effect on total, conduit and peripheral flow waveforms (Figure 1(b,f,h)).

We cannot measure conduit and peripheral pressures *in vivo*, but we can approximate them through the reservoir and excess pressures, respectively 56, which can be calculated from a pressure waveform measured at an arbitrary location in arteries 66. This approximation improves with the reflection coefficients for forward-travelling waves at junctions 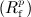 given by Equation (12) approaching zero 56. Thus, reservoir and excess pressures in normal conditions and under the effect of pharmacological drugs affecting vessel compliance and resistance could be used for an *in vivo* verification of the numerical results presented in this section.

### 3.11 Sensitivity of the modified wave intensity profile to sampling frequency and errors in pulse wave velocity estimate

We analyse the sensitivity of the modified forward and backward wave intensity profiles (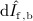), transit time (TT) and apparent reflection coefficient (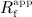) in the midpoint of the single-vessel aortic model to decreasing sampling frequency (Section 3.11.1) and increasing errors in the estimate of PWV (Section 3.11.2).

#### 3.11.1 Sampling frequency

**Figure 12:**
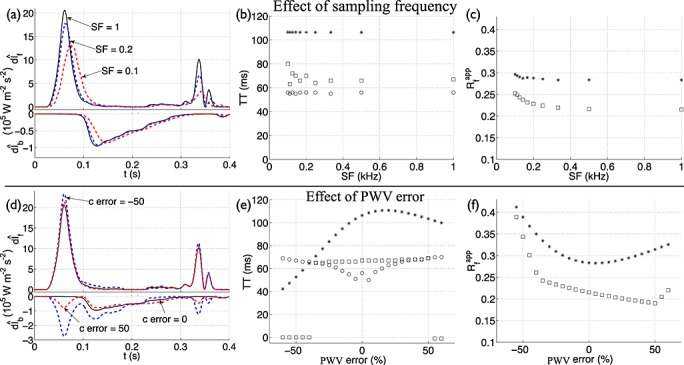
(a,d) Forward 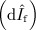 and backward 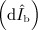 components of modified wave intensity with time in the midpoint of the single-vessel aortic model coupled to a matched RCR Windkessel outflow model (Figure 3(b)). They were calculated using the original model (black solid lines), with *c* at diastolic pressure, and changing the (a) sampling frequency (SF in kHz) or (d) error in the PWV relative to the theoretical PWV (*c* error in %), as indicated by the labels (red and blue dashed lines). In the original model, we have SF = 1 kHz and *c* error = 0%. Note the different scaling of 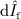 and 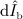. Effect of (b,c) SF and (e,f) PWV error on the transit time (TT) and apparent reflection coefficient 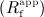 calculated using foot (circles), peak (squares) or area-average (stars) values, as illustrated in Figure 5.

The forward 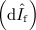 and backward 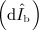 wave intensity profiles change with decreasing sampling frequency of pressure and flow velocity (Figure 12(a)). From 1 to 0.2 kHz, the peak values of 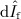 and 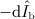 change by less than 15%, the errors in the TT estimates given by foot values remain below 10% relative to the theoretical TT = 51 ms (Figure 12(b)), and the calculated 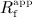 using peak and area-average values change by less than 6% relative to the corresponding values at 1 kHz (Figure 12(c)). We observed greater differences in 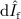 and 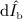 at 0.1 kHz (Figure 12(a)) and below, which lead to greater relative errors in TT and 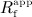 (Figure 12(b,c)).

Furthermore, we observed a drop in the peak of the valve wave (described in Section 3.5) with decreasing sampling frequency; this wave is small at 0.2 kHz and absent at 0.1 kHz (Figure 2(a)). This result is in agreement with aortic *in vivo* data showing that the valve wave is small or absent in the traditional wave intensity profile for sampling frequencies below 0.2 kHz 2,4,15 and present for greater frequencies 21,57–59.

Our results suggest a small sensitivity of the wave intensity profile to pressure and velocity data sampled at 0.2 kHz or above, assuming data free of any other error, and support the use of foot values of 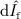 and 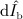 to estimate the TT to a dominant reflection site. However, sampling frequencies greater than 0.2 kHz may be necessary at higher heart rates and under strong myocardial contractions.

#### 3.11.2 Pulse wave velocity error

Introducing an error in the PWV given by Equation (5) at diastolic area when calculating 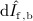 results in a backward compression wave developing at the same time as the (incident) LV-contraction wave (Figure 12(d)). The amplitude of this fictitious wave increases with increasing PWV error, which leads to increasingly greater changes in the estimated TT (Figure 12(e)) and 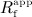 (Figure 12(f)) with respect to their values free of PWV error. A PWV error of 20% changes the estimates of TT and 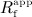 by less than 7% using peak values and 16% using area-average values, relative to the corresponding values free of error. Greater PWV errors yield dramatic changes in the calculated TT and 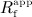 using peak values, because the peak value of 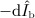 is attained in the fictitious wave instead of the genuine reflected wave (Figure 12(d)). Lastly, using the local minimum between the fictitious and genuine reflected waves to determine the foot of the reflected wave, the TT estimates given by foot values are more sensitive to PWV errors within ± 20% than those given by peak values (Figure 12(e)).

Our results suggest a small variability of 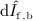 in the aorta using estimates of PWV with errors up to ± 20%, in agreement with results reported for traditional d*I*_f,b_ using *in vivo* measurements in human coronary arteries 67. In Section 3.8, we showed that estimates of local PWV at the ascending aorta with errors smaller than 20% are feasible from simultaneous pressure and flow velocity measurements using the modified 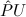
–loop method.

## 4 CONCLUSIONS

We have presented a novel analysis of arterial pulse wave propagation that combines traditional WIA with identification of Windkessel pressures to account for the effect on the pressure waveform of peripheral wave reflections. Our modified wave intensity profiles allow us to study the predominant waves that shape pressure and flow waveforms during systole, starting from a ‘true’ reflection-free period in early systole. The Windkessel pressure allows us to (i) quantify the contribution to the pressure waveform over the whole cardiac cycle of reflected waves originating in previous cardiac cycles and (ii) study the buffering effect of the vasculature, which depends on vessel compliances, peripheral resistances, outflow pressures and the flow at the root (Section 3.9).

We have shown that traditional WIA identifies the timing, direction and magnitude of the predominant waves that shape aortic pressure and flow waveforms in systole (Section 3.1 to 3.5) but fails to identify the important contribution to the pressure waveform of peripheral reflections. These reflections persist for several cardiac cycles and make up most of the pressure waveform, especially in diastole and at the start of cardiac contraction (Section 3.6). Ignoring the contribution of peripheral reflections to the pressure waveform leads to an erroneous indication of a reflection-free period in early systole and additional error in the estimates of (i) PWV at the ascending aorta given by the *PU*–loop method (9.5% error using rabbit *in vivo* data) and (ii) TT to a dominant reflection site calculated from the wave intensity profile (27% error using numerical data). These errors decreased to 1.3% and 10%, respectively, when peripheral reflections were considered in the calculations using the Windkessel pressure (Section 3.8 and 3.9.1).

We have used our new analysis of wave propagation to study the effects of vessel compliance and peripheral resistance on numerically-generated aortic pressure (*P*) and flow (*Q*) waveforms (Section 3.10). With decreasing compliance, the pulse pressure increases, a smaller portion of *P* is made up of reflected waves originating from earlier cycles, there is less damping of *Q*, and pulse wave energy increases, suggesting that the LV must produce more energy to propel the same amount of blood flow throughout a stiffer vasculature. With increasing resistance, the mean pressure raises (but not the pulse pressure), a greater portion of *P* is made up of reflected waves originating from earlier cycles, and there is little change in *Q* and wave energy. We have also shown that vessel compliance has a similar effect on reflected waves originating from internal junctions, aortic root and tapered vessels to those originating from the periphery, whereas peripheral resistances only affect reflected waves originating from the periphery.

Lastly, our results suggest a small sensitivity of the forward and backward wave intensity profiles to pressure and velocity data sampled at 0.2 kHz or above or to errors in the estimate of PWV within ± 20%, assuming data free of any other error.

It is important to note that our modified WIA differs from the *reservoir-wave hypothesis* 55,56,66, which has been shown not to be beneficial for WIA 21. In this hypothesis, all the Windkessel pressure is separated from the measured pressure waveform, whereas our new method separates only the Windkessel pressure from previous cardiac cycles. The latter allows us to do WIA on all components of the pressure waveform generated in the current cardiac cycle, starting from a ‘true’ reflection-free period.

## Figures and Tables

**Figure A.1:**
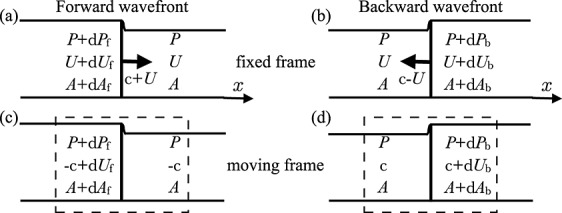
Changes in area (d*A*), flow velocity (d*U*) and pressure (d*P*) across a pulse wavefront travelling (a,c) forward (in the positive *x* direction) and (b,d) backward with speeds of *c* + *U* and *c* − *U*, respectively, where *c* is the local pulse wave velocity. They are measured from a frame of reference fixed to the vessel (a,b) and moving with the wavefront (c,d). The dashed rectangles indicate the control volume used to derive Equations (A.2)–(A.7).

**Figure C.1:**
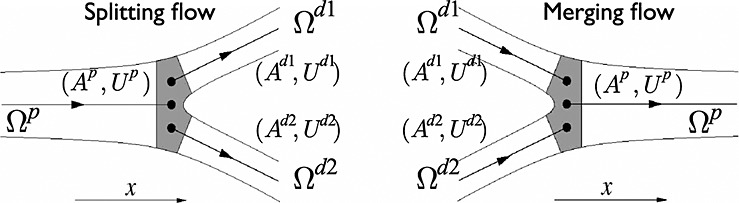
Nomenclature for the two types of junctions considered, splitting (left) and merging (right) flows. The arrows indicate the positive direction of the axial coordinate *x*.

## Footnotes

‡ In this article, the term ‘wave’ refers to a change in blood pressure, flow and luminal area with a finite duration; ‘wavefront’ refers to infinitesimal changes in these properties; and ‘waveform’ refers to the shape of these properties over the cardiac cycle. Wavefronts are the elemental waves in wave intensity analysis (WIA).

§ This is difficult to observe in Figures 1(a,b) and 3(a,d) because the decrease in mean pressure is small compared to pulse pressure.

¶ The mean flow does not decrease in the single-vessel aortic model because the outlet is the only outflow of this model.

## APPENDIX A: DERIVATION OF THE WATER HAMMER EQUATIONS

Following 15, we consider the area (*A*), velocity (*U*) and pressure (*P*) waveforms to be made of successive wavefronts, which travel through the arterial network propagating changes in *A*, *U* and *P*. Across an individual wavefront, we define the changes in area, velocity and pressure as d*A*, d*U* and d*P*, respectively. We take the linear assumption that these changes can be separated into changes across the forward-travelling ( d*A*_f_, d*U*_f_, d*P*_f_) and backward-travelling ( d*A*_b_, d*U*_b_, d*P*_b_) wavefronts (Figure A.1), that is

A.1

We define the forward direction as the direction of mean blood flow, in which *x* increases.

Applying conservation of mass in the control volume for the frame moving with the forward-travelling wavefront (Figure 13(c)) yields , where *ρ* is the density of blood assumed to be constant and *c* is the local PWV. This expression reduces to

A.2

ignoring the term . Applying conservation of momentum to the same moving frame yields , which reduces to

A.3

ignoring terms with products of d*A*_f_, d*U*_f_ and d*P*_f_. Replacing *c* d*A*_f_ in (A.3) with *A* d*U*_f_ from (A.2) yields the water hammer equation . Replacing *A* d*U*_f_ in (A.3) with *c* d*A*_f_ from (A.2) yields

A.4

Similarly, conservation of mass and momentum in the control volume for the frame moving with the backward-travelling wavefront (Figure 13(d)) yields the other water hammer equation ,

A.5

A.6

Combining Equations (A.4) and (A.6), using  and , yields

A.7

## APPENDIX B: CALCULATION OF THE PULSE WAVE VELOCITY

Equations (8), (A.2) and (A.7) allow us to calculate the local PWV (*c*) at an arbitrary location in the arteries from simultaneous measurements of *P* and *U*, *A* and *U*, and *P* and *A*, respectively, using the methods described in the following two subsections. In all these methods, *c* is assumed to be constant; that is *c* = *c*(*x*), and d*A*, d*U* and d*P* are calculated as the change in the measured *A*, *U* and *P*, respectively, in a sampling period d*t* (e.g. d*A*(*t*) = *A*(*t* + d*t*) − *A*(*t*)) using a Savitzky–Golay filter 16,51,52 ,p. 650.

### From simultaneous measurements of *P* (or *A*) and *U* using ‘loop’ methods

The ‘loop’ methods rely on the existence of a period within the cardiac cycle in which the plots of *P* versus *U* (*PU*–loop) and ln *A* versus *U* (ln*AU*–loop) are approximately linear. In normal physiological conditions, this linearity has been observed in early systole in the *PU*–loop of the human aorta and main pulmonary artery 16,53 and ln*AU*–loop of the human carotid artery 68, which suggests that d*A*, d*U* and d*P* in early systole are predominantly made of wavefronts propagating towards distal locations. Thus, d*A* = d*A*_f_, d*U* = d*U*_f_ and d*P* = d*P*_f_ are reasonable assumptions and Equations (8) and (A.2) yield, respectively,

B.1

B.2

They allow us to calculate *c* from the slope of the (approximately) linear part in the *PU*– and ln*AU*–loops; this slope is *ρc* and 1/*c*, respectively. In the case of the *PU*–loop, we need to know the density of blood (*ρ*).

In Section 3.8, we showed that elimination of the Windkessel pressure generated in previous cardiac cycles from *P* improves the accuracy of the value of *c* given by the *PU*–loop method in the ascending aorta.

### From simultaneous measurements of *P* and *A* using a new analytical expression

According to Equation (A.7), (d*P*)^2^ = *ρ*^2^*c*^4^ (d(ln *A*))^2^. Adding the terms on the left and right of this equation over a cardiac cycle, assuming *ρ* and *c* to be constant, yields

B.3

This equation is similar in form to the so-called ‘single-point’ method 16,69 for simultaneous *P* and *U* measurements, though Equation (B.3) follows directly from conservation of mass and momentum and does not require minimising wave energy over the cardiac cycle as does the ‘single-point’ method.

## APPENDIX C: WAVEFRONT REFLECTIONS AT JUNCTIONS

Consider three arterial segments Ω^*j*^, *j* = *p*,*d*1,*d*2, joining in a splitting or merging flow junction (Figure C.1). Three perturbations  of the initial states , *j* = *p*,*d*1,*d*2, of luminal cross-sectional area, elastic component of pressure and flow rate, respectively, propagating towards the junction (along each corresponding segment Ω^*j*^) will produce a new wave in each segment, denoted by , *j* = *p*,*d*1,*d*2, which will propagate away from the junction. This appendix shows how these three new waves can be calculated using the linearised 1-D equations,

C.1

where *a*, *p*_e_ and *q* are the perturbation variables for area, elastic component of pressure and flow rate, respectively, that is , and

C.2

are the elastic wall compliance, fluid inertia and viscous fluid resistance, respectively, per unit length of vessel. Equations (C.1) follow from linearising (1) and (2) about the reference state (*A*,*P*,*P*_e_,*Q*) = (*A*_0_,0,0,0), with Γ = 0 and *β* and *A*_0_ constant along *x* 39,54.

Conservation of mass and continuity of the total pressure at the junction yield

C.3

C.4

In addition, the linear characteristic variables‖, , propagating towards the junction remain unchanged after the reflection. Thus, for a splitting flow junction (Figure 14, left) we have

C.5

where , *j* = *p*,*d*1,*d*2, is the characteristic impedance of segment Ω^*j*^ and  is the pulse wave velocity at zero blood pressure. Moreover,

C.6

because the initial characteristic variables moving away from the junction are all zero. Combination of Equations (C.5) and (C.6) yields

C.7

Substitution of these expressions into Equation (C.3) and using Equation (C.4) leads to

C.8

where , *j* = *p*,*d*1,*d*2, is the characteristic admittance of segment Ω^*j*^.

If we perturb one segment at a time with , *j* = *p*,*d*1,*d*2, so that  for *i* ≠ *j*, then from Equation (C.8) we have

C.9

Defining the reflection coefficient , *j* = *p*,*d*1,*d*2, as the ratio of the change of pressure across the reflected wave to the change of pressure in the incident wave; that is , *j* = *p*,*d*1,*d*2, and using Equation (C.9) to write  as a function of  yields Equation (12).

For a merging flow junction (Figure 14, right), Equations (C.3) and (C.4) are also valid, whereas Equations (C.5) and (C.6), respectively, become

C.10

C.11

Combining Equations (C.3), (C.4), (C.10) and (C.11) as described previously for the splitting flow case also yields Equation (12).

Note that from  in (C.1), we have  and , *j* = *p*,*d*1,*d*2. Thus, , *j* = *p*,*d*1,*d*2, is satisfied, which shows that the reflection coefficients also give the ratio of the change of area across the reflected wave to the change of area in the incident wave. However, in terms of changes in the flow rates, we have , *j* = *p*,*d*1,*d*2, which follows from transforming *δq*^*j*^ and Δ*q*^*j*^ into pressures using Equations (C.6) and (C.7) for the splitting flow case and Equations (C.10) and (C.11) for the merging flow case. Defining the changes in flow velocity as  and , we have , *j* = *p*,*d*1,*d*2.

## Supporting Information

Supporting info item
