# Supplementary material for: Novel wave intensity analysis of arterial pulse wave propagation accounting for peripheral reflections
Source: Int J Numer Method Biomed Eng. 2013 Oct 16;30(2):249–79. doi: 10.1002/cnm.2602 (PMC4297358; doi:10.1002/cnm.2602)
Supplement: Supplementary file 1 — Supporting info item [file cnm0030-0249-sd1.pdf]

INTERNATIONAL JOURNAL FOR NUMERICAL METHODS IN BIOMEDICAL ENGINEERING
Int. J. Numer. Meth. Biomed. Engng. (Supp. Mat.) 2013; 1–5
Published online in Wiley InterScience (www.interscience.wiley.com). DOI: 10.1002/cnm.2602

Novel wave intensity analysis of arterial pulse wave propagation accounting for peripheral reflections (supplementary material)

Jordi Alastruey[1,*], Anthony A.E. Hunt[2], Peter D. Weinberg[2]

[1]*Department of Biomedical Engineering, Division of Imaging Sciences and Biomedical Engineering, King's College London, King's Health Partners, St. Thomas' Hospital, London, SE1 7EH, U.K.*
[2]*Department of Bioengineering, Imperial College, London, SW7 2AZ, U.K.*

1. EFFECT OF VESSEL COMPLIANCE AND PERIPHERAL RESISTANCE ON TRANSIT TIME AND APPARENT REFLECTION COEFFICIENT IN THE PRESENCE OF A SINGLE PERIPHERAL REFLECTION SITE

We will use the single-vessel aortic model coupled to a matched RCR Windkessel outflow model (Fig. 3b) to show that, in the presence of a single peripheral reflection site, the new forward ($\mathrm{d}\hat{I}_\mathrm{f}$) and backward ($\mathrm{d}\hat{I}_\mathrm{b}$) wave intensity profiles allow us to estimate theoretical changes in transit time (TT) due to varying vessel compliance and peripheral resistance (Section 1.1). We also provide numerical results on the effect of varying vessel compliance and peripheral resistance on the apparent reflection coefficient ($R_\mathrm{f}^\mathrm{app}$) (Section 1.2).

1.1. Transit Time

Calculation of the TT using Eq. (16) in the midpoint of the aortic model with several values of the Young's modulus of the arterial wall (E), from 0.2 MPa to 0.8 MPa, shows a decrease in TT with increasing E (Fig. 1c, supp. mat.) and, hence, decreasing vessel compliance. According to Eq. (5), the theoretical pulse wave velocity (PWV) decreases by 28% with half the original $E = 0.4$ MPa and increases by 41% with double the original E; that is, pulse wavefronts travel at a greater speed in stiffer vessels. Given that the distance to the peripheral reflection site is constant in our aortic model, the corresponding changes in theoretical TT are +39% and -29%. The changes in TT given by Eq. (16) are 38% and -25% using foot values of $\mathrm{d}\hat{I}_\mathrm{f}$ and $\mathrm{d}\hat{I}_\mathrm{b}$, 19% and -16% using peak values, and 6% and -9% using area-average values. These results support the use of foot values, rather than peak or area-average values, when calculating the TT to a dominant reflection site using our modified WIA.

Furthermore, the TT given by Eq. (16) in the midpoint of the aortic model decreases with increasing peripheral resistance (R_T) (Fig. 1f, supp. mat.), since the increase in pressure associated with the increase in R_T yields greater luminal area (A) and, hence, greater PWV, according to Eq. (5). Changes in the theoretical TT with R_T are also better captured by TT estimates given by Eq. (16) using foot values than peak or area-average values.

The results in this section show the ability of our new WIA to estimate the transit time to a dominant peripheral reflection site, using foot values of wave intensity $\mathrm{d}\hat{I}_\mathrm{f,b}$ to calculate the times of the incident and reflected waves.

*Correspondence to: Jordi Alastruey, Department of Biomedical Engineering, Division of Imaging Sciences and Biomedical Engineering, King's College London, King's Health Partners, St. Thomas' Hospital, London, SE1 7EH, U.K.
E-mail: jordi.alastruey-arimon@kcl.ac.uk

© 2013 The Authors. *International Journal of Numerical Methods in Biomedical Engineering* published by John Wiley & Sons, Ltd.

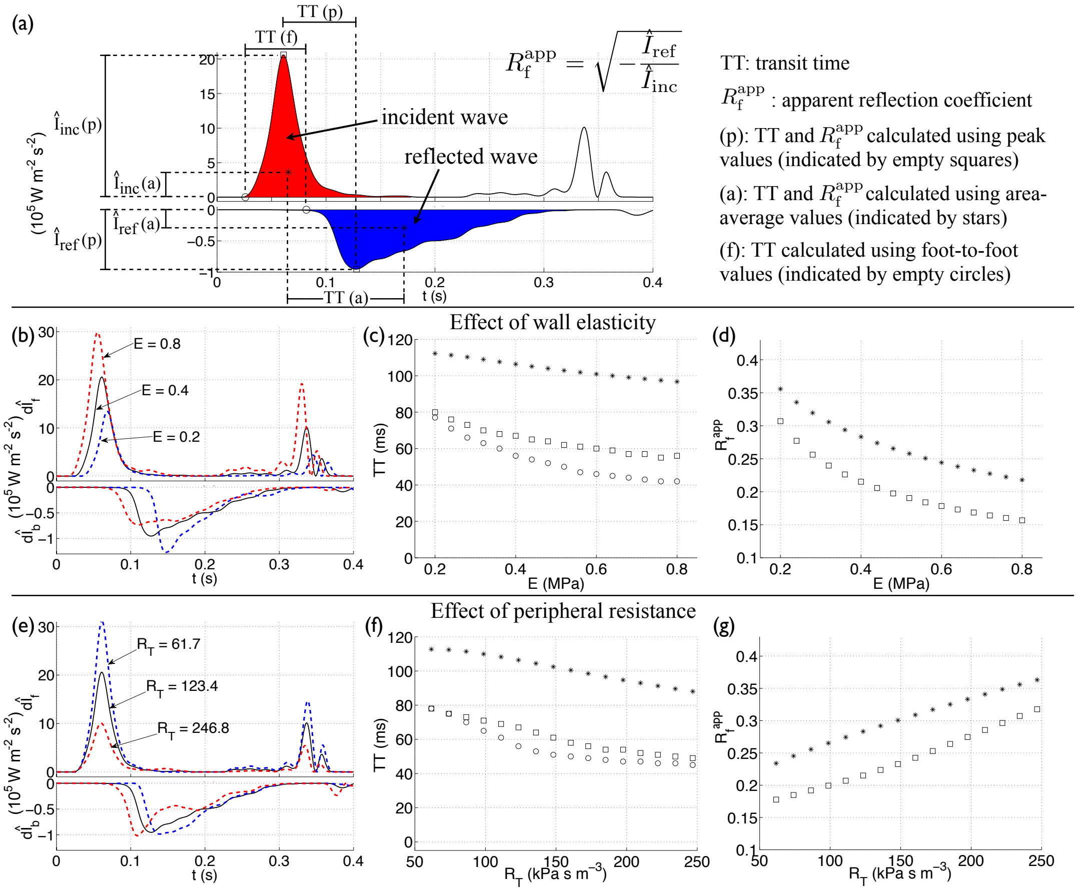

Figure 1. (a) Illustration of the calculation of the transit time (TT) between the incident and reflected waves and apparent reflection coefficient (R_f^{app}) using the new wave intensity profiles $d\hat{I}_{f,b}$. (b,e) Forward ($d\hat{I}_f$) and backward ($d\hat{I}_b$) components of modified wave intensity with time in the midpoint of the single-vessel aortic model coupled to a matched RCR Windkessel outflow model (Fig. 3b). They were calculated using the original model (black solid lines), with c at diastolic pressure, and changing the (b) wall elastic modulus (E in MPa) or (e) peripheral resistance (R_T in kPa s m^{-3}) as indicated by the labels (red and blue dashed lines). In the original model we have $E = 0.4$ MPa and $R_T = 123.4$ kPa s m^{-3}. Note the different scaling of $d\hat{I}_f$ and $d\hat{I}_b$. Effect of (c,d) E and (f,g) R_T on TT and R_f^{app} calculated using foot (circles), peak (squares) or area-average (stars) values, as illustrated in (a).

1.2. Apparent Reflection Coefficient

The estimate of R_f^{app} given by Eq. (16) in the midpoint of our aortic model decreases with increasing E (Fig. 1d, supp. mat.), since the amplitude of the incident wave raises and the amplitude of the reflected wave drops with increasing E (Fig. 1b, supp. mat.). Changes in R_T affect the amplitude of the incident wave, but produce little changes in the amplitude of the reflected wave (Fig. 1e, suppl. mat.). The incident amplitude decreases with increasing R_T, since the increase in A with R_T yields smaller flow velocities (U) and, hence, smaller wavefronts dU contributing to $d\hat{I}_f$. As a result, the R_f^{app} increases with increasing R_T (Fig. 1g, suppl. mat.).

© 2013 The Authors. *International Journal of Numerical Methods in Biomedical Engineering* published by John Wiley & Sons, Ltd.
Prepared using cnmauth.cls

DOI: 10.1002/cnm.2602

2. NOMENCLATURE AND ABBREVIATIONS

Table I. Nomenclature, abbreviations and SI units. Units commonly used in the clinic that differ from SI units are shown after a semicolon.

1-D		one-dimensional
A	(m^2; cm^2 or mm^2)	area of the luminal cross section of the vessel
A_0	(m^2)	luminal cross-sectional area at zero blood pressure
a	(m^2)	linear perturbation for the luminal cross-sectional area
CCA		common carotid artery
CO	($m^3\ s^{-1}$; ml s^{-1}, l min^{-1})	cardiac output generated by the left ventricle
C-R	(m)	rabbit crown-to-rump length
C	(m^3 Pa^{-1})	compliance of the peripheral RCR Windkessel model
C_{0D}	(m^3 Pa^{-1})	compliance of an arterial segment
C_{1D}	(m^2 Pa^{-1})	compliance per unit length of arterial segment
C_T	(m^3 Pa^{-1}; ml $mmHg^{-1}$)	total systemic compliance
C_c, C_p	(m^3 Pa^{-1})	total conduit or peripheral compliance, respectively
C_f, C_b		forward or backward characteristic path, respectively
c	(m s^{-1})	pulse wave velocity
c_0	(m s^{-1})	pulse wave velocity at zero blood pressure
c_{AU}	(m s^{-1})	pulse wave velocity calculated using the lnAU–loop method
c_{PA}	(m s^{-1})	pulse wave velocity calculated from simultaneous pressure and area measurements
c_{PU}	(m s^{-1})	pulse wave velocity calculated using the PU–loop method
$\hat{c}_{PU}$	(m s^{-1})	pulse wave velocity calculated using the modified $\hat{P}U$–loop method
c_{ff}	(m s^{-1})	pulse wave velocity calculated using the 'foot-to-foot' method
DG		discontinuous Galerkin
$\mathrm{d}A$	(m^2)	change in luminal area across a wavefront
$\mathrm{d}A_f$, $\mathrm{d}A_b$	(m^2)	change in luminal area across a forward or backward wavefront, respectively
$\mathrm{d}I$	(W m^{-2})	traditional wave intensity
$\mathrm{d}\hat{I}$	(W m^{-2})	new wave intensity cleared of contributions of waves reflected in previous cardiac cycles
$\mathrm{d}I_f$, $\mathrm{d}I_b$	(W m^{-2})	traditional wave intensity of a forward or backward wavefront, respectively
$\mathrm{d}\hat{I}_f$, $\mathrm{d}\hat{I}_b$	(W m^{-2})	new wave intensity of a forward or backward wavefront, respectively, cleared of contributions of waves reflected in previous cardiac cycles
$\mathrm{d}I_{inc}$, $\mathrm{d}I_{ref}$	(W m^{-2})	wave intensity of an incident or reflected wavefront, respectively
$\mathrm{d}I_s$	(W m^{-2})	wave intensity during early systolic ejection
$\mathrm{d}I_w$	(W m^{-2})	wave intensity in diastole given by the Windkessel pressure and flow
$\mathrm{d}P$	(Pa)	change in blood pressure across a wavefront
$\mathrm{d}\hat{P}$	(Pa)	change in blood pressure across a wavefront cleared of contributions of waves reflected in previous cardiac cycles
$\mathrm{d}P_f$, $\mathrm{d}P_b$	(Pa)	traditional change in blood pressure across a forward or backward wavefront, respectively
$\mathrm{d}\hat{P}_f$, $\mathrm{d}\hat{P}_b$	(Pa)	change in blood pressure across a forward or backward wavefront, respectively, cleared of contributions of waves reflected in previous cardiac cycles
$\mathrm{d}P_{inc}$, $\mathrm{d}P_{ref}$	(Pa)	change in blood pressure in the incident or across the reflected wavefront, respectively
$\mathrm{d}P_s$	(Pa)	change in blood pressure across a wavefront during early systolic ejection
$\mathrm{d}p_w$	(Pa)	change in p_w across a wavefront during diastole
$\mathrm{d}q_w$	($m^3\ s^{-1}$)	change in q_w across a wavefront during diastole
$\mathrm{d}U$	(m s^{-1})	change in blood flow velocity across a wavefront

© 2013 The Authors. *International Journal of Numerical Methods in Biomedical Engineering* published by John Wiley & Sons, Ltd.

DOI: 10.1002/cnm.2602

Table II. Continuation of nomenclature, abbreviations and units.

$\mathrm{d}U_\mathrm{f}$, $\mathrm{d}U_\mathrm{b}$	(m s^{-1})	traditional change in blood flow velocity across a forward or backward wavefront, respectively
$\mathrm{d}\hat{U}_\mathrm{f}$, $\mathrm{d}\hat{U}_\mathrm{b}$	(m s^{-1})	change in blood flow velocity across a forward or backward wavefront, respectively, cleared of contributions of waves reflected in previous cardiac cycles
$\mathrm{d}U_\mathrm{inc}$, $\mathrm{d}U_\mathrm{ref}$	(m s^{-1})	change in blood flow velocity in the incident or across the reflected wavefront, respectively
$\mathrm{d}U_\mathrm{s}$	(m s^{-1})	change in blood flow velocity across a wavefront during early systolic ejection
$\mathrm{d}W_\mathrm{f}$, $\mathrm{d}W_\mathrm{b}$	(m s^{-1})	change in forward or backward characteristic variable, respectively
$\mathrm{d}t$	(s)	time between two adjacent sampling points of P or U
ECG		electrocardiogram
E	(Pa)	Young's modulus of the arterial wall
f	(N m^{-1})	frictional force per unit length of vessel
HR	(s^{-1}; beats min^{-1})	heart rate
h	(m; mm)	wall thickness of the artery
I_inc, I_ref	(W m^{-2})	magnitude of the incident or reflected wave, respectively, of the wave intensity profile
$\hat{I}_\mathrm{inc}$, $\hat{I}_\mathrm{ref}$	(W m^{-2})	magnitude of the incident or reflected wave, respectively, of the wave intensity profile cleared of contributions of waves reflected in previous cardiac cycles
LV		left ventricle
L_1D	(kg m^{-5})	blood inertia per unit length of vessel
l	(m; cm)	length of an arterial segment
M1		normal young 55-artery model
M2		normal old 55-artery model
M3		hypertensive young 55-artery model
M4		hypertensive old 55-artery model
$M-1$		total number of terminal arterial segments in a distributed 1-D model
N		total number of arterial segments in a distributed 1-D model
PP	(Pa; mmHg)	pulse pressure; that is, difference between the maximum, systolic, and minimum, diastolic, pressures
PWV	(m s^{-1})	pulse wave velocity
P	(Pa; mmHg)	blood pressure averaged over the luminal cross section
$\hat{P}$	(Pa)	blood pressure cleared of contributions of waves reflected in previous cardiac cycles
P_0	(Pa)	reference blood pressure for the calculation of forward and backward pressure components
P_con, P_per	(Pa)	conduit or peripheral component of blood pressure, respectively
P_d	(Pa; mmHg)	diastolic pressure
P_e	(Pa)	elastic component of blood pressure
P_f, P_b	(Pa; mmHg)	traditional forward or backward component of blood pressure, respectively
$\hat{P}_\mathrm{f}$, $\hat{P}_\mathrm{b}$	(Pa)	forward or backward component of blood pressure, respectively, cleared of contributions of waves reflected in previous cardiac cycles
P_m	(Pa; mmHg)	mean blood pressure
P_out	(Pa; mmHg)	blood pressure at which flow to the microcirculation ceases
p_e	(Pa)	linear perturbation for the elastic component of blood pressure
p_w	(Pa)	space-independent Windkessel blood pressure
Q	(m^3 s^{-1}; ml s^{-1}, l min^{-1})	blood volume flow rate
Q_con, Q_per	(m^3 s^{-1})	conduit or peripheral component of the flow rate, respectively
Q_in	(m^3 s^{-1}; l min^{-1})	blood volume flow rate at the aortic root
q	(m^3 s^{-1})	linear perturbation for the blood volume flow rate
q_in, q_out	(m^3 s^{-1})	linear perturbation of the volume flow rate at the inlet or outlet, respectively, of an arterial segment
q_w	(m^3 s^{-1})	blood volume flow rate driven by $p_\mathrm{w} - P_\mathrm{out}$
R_1, R_2	(Pa s m^{-3})	inflow or outflow resistance, respectively, of the peripheral RCR Windkessel model

© 2013 The Authors. *International Journal of Numerical Methods in Biomedical Engineering* published by John Wiley & Sons, Ltd.

DOI: 10.1002/cnm.2602

Table III. Continuation of nomenclature, abbreviations and units.

R_{1D}	(Pa s m^{-4})	resistance per unit length of vessel due to blood viscosity
R_{T}	(Pa s m^{-3}; mmHg s ml^{-3})	net peripheral resistance of the arterial network
R_{f}		reflection coefficient for a pulse wavefront at a local site of impedance mismatch
$R_{\mathrm{f}}^{\mathrm{app}}$		apparent reflection coefficient calculated from the incident and reflected waves of the wave intensity profile
$R_{\mathrm{f}}^{p,d1,d2}$		reflection coefficient of a wavefront propagating respectively in the parent, first daughter or second daughter vessels joining in an arterial junction
r	(m; cm)	radius of the luminal cross section of the vessel
r_{d}	(m; cm)	luminal radius at diastolic pressure
SEM		standard error of the mean
SF	(s^{-1}; Hz, kHz)	sampling frequency
TT	(s; ms)	transit time between the incident and reflected waves of the wave intensity profile
T_0	(s)	reference time in the calculation of the space-independent Windkessel blood pressure p_{w}
$\mathcal{T}_{\mathrm{inc}}, \mathcal{T}_{\mathrm{ref}}$	(s)	time of arrival of the incident or reflected wave, respectively, in the wave intensity profile
t	(s)	time
$t_{\mathrm{ini}}, t_{\mathrm{end}}$	(s)	time at the start or end, respectively, of a wave in the wave intensity profile
U	(m s^{-1}; cm s^{-1})	axial blood flow velocity averaged over the luminal cross section
U_0	(m s^{-1})	reference average flow velocity for the calculation of forward and backward velocity components
$U_{\mathrm{inc}}, U_{\mathrm{ref}}$	(m s^{-1})	average flow velocity during the propagation of an incident or reflected wavefront, respectively
$U_{\mathrm{f}}, U_{\mathrm{b}}$	(m s^{-1}; cm s^{-1})	traditional forward or backward component of blood velocity, respectively
$\hat{U}_{\mathrm{f}}, \hat{U}_{\mathrm{b}}$	(m s^{-1}; cm s^{-1})	forward or backward component of blood velocity, respectively, cleared of contributions of waves reflected in previous cardiac cycles
WIA		wave intensity analysis
$W_{\mathrm{f}}, W_{\mathrm{b}}$	(m s^{-1})	nonlinear forward or backward characteristic variable, respectively
$w_{\mathrm{f}}, w_{\mathrm{b}}$	(m^3 s^{-1})	linear forward or backward characteristic variable, respectively
x	(m)	axial coordinate of an arterial segment Ω
$\hat{x}(t)$		parametric function in the (x,t) space
Y_0	(m^3 Pa^{-1} s^{-1})	characteristic admittance of the vessel
Z_0	(Pa s m^{-3})	characteristic impedance of the vessel
β	(Pa m)	parameter related to the elastic tone of the vessel wall
Γ	(Pa s m)	parameter related to the viscosity of the vessel wall
Δa	(m^2)	linear perturbation of luminal cross-sectional area propagating toward a junction
Δp_{e}	(Pa)	linear perturbation of elastic component of pressure propagating toward a junction
Δq	(m^3 s^{-1})	linear perturbation of flow rate propagating toward a junction
Δu	(m s^{-1})	linear perturbation of flow velocity propagating toward a junction
δa	(m^2)	linear perturbation of luminal cross-sectional area propagating away from a junction
δp_{e}	(Pa)	linear perturbation of elastic component of pressure propagating away from a junction
δq	(m^3 s^{-1})	linear perturbation of flow rate propagating away from a junction
δu	(m s^{-1})	linear perturbation of flow velocity propagating away from a junction
μ	(Pa s)	dynamic viscosity of blood
ρ	(kg m^{-3})	blood density
φ	(Pa s)	viscosity of the vessel wall
Ω		1-D domain of an arterial segment

© 2013 The Authors. *International Journal of Numerical Methods in Biomedical Engineering* published by John Wiley & Sons, Ltd.
